# An improved gray wolf optimization to solve the multi-objective tugboat scheduling problem

**DOI:** 10.1371/journal.pone.0296966

**Published:** 2024-02-26

**Authors:** Peng Yao, Xingfeng Duan, Jiale Tang

**Affiliations:** College of Navigation, Jimei University, Xiamen, Fujian, China; Istinye University: Istinye Universitesi, TURKEY

## Abstract

With the continuous prosperity of maritime transportation on a global scale and the resulting escalation in port trade volume, tugboats assume a pivotal role as essential auxiliary tools influencing the ingress and egress of vessels into and out of ports. As a result, the optimization of port tug scheduling becomes of paramount importance, as it contributes to the heightened efficiency of ship movements, cost savings in port operations, and the promotion of sustainable development within the realm of maritime transportation. However, a majority of current tugboat scheduling models tend to focus solely on the maximum operational time. Alternatively, the formulated objective functions often deviate from real-world scenarios. Furthermore, prevailing scheduling methods exhibit shortcomings, including inadequate solution accuracy and incompatibility with integer programming. Consequently, this paper introduces a novel multi-objective tugboat scheduling model to align more effectively with practical considerations. We propose a novel optimization algorithm, the Improved Grey Wolf Optimization (IGWO), for solving the tugboat scheduling model. The algorithm enhances convergence performance by optimizing convergence parameters and individual updates, making it particularly suited for solving integer programming problems. The experimental session designs several scale instances according to the reality of the port, carries out simulation experiments comparing several groups of intelligent algorithms, verifies the effectiveness of IGWO, and verifies it in the comprehensive port area of Huanghua Port to get the optimal scheduling scheme of this port area, and finally gives management suggestions to reduce the cost of tugboat operation through sensitivity analysis.

## 1. Introduction

Against the backdrop of open world ports and economic development, international trade competition has become increasingly fierce. The expansion of port scale and the continuous growing operational demands have become major trends and currents [1]. The demand for tugboats during ship arrivals and departures at ports is increasing. At the same time, sustainable development and low-carbon environmental issues in maritime transportation have become hot topics of concern [2,3].

Tugboats are crucial for components in maintaining port operations, are costly and subject to increasing wear and tear, while port resources are limited [4]. As a result, optimizing tugboat scheduling has become a crucial aspect to reduce port operating costs [5]. By optimizing the tugboat scheduling process reasonably, a significant amount of tugboat resources can be saved, accelerating the efficiency of port vessel operations and reducing carbon emissions to a certain extent, thereby promoting energy conservation and environmental protection within ports.

### 1.1. Literature review

The tugboat scheduling problem (Tug-SP) originated from the Job-shop Scheduling Problem (JSP). Traditional tugboat scheduling models mainly rely on the experience of scheduling personnel, supplemented by some integer programming methods [6–8]. This kind of scheduling model has more advantages in solving small-scale scheduling, but with the continuous growth of port operational demands and scale, traditional methods have become inadequate, necessitating more scientific and effective approaches to solve the tugboat scheduling problem. Current research focuses on establishing tugboat scheduling models that are in line with practical situations and employing suitable metaheuristic algorithms for solution.

Existing research related to tugboat scheduling can be broadly categorized into two phases, as shown in Table 1. In the first stage, researchers mainly concentrated in the initial exploration of intelligent tugboat scheduling research, placing greater emphasis on model construction while neglecting the actual needs of ports. For example, researchers such as Liu, Xu, and Kang described Tug-SP as a multiprocessor task scheduling problem, optimized the port tug tugboat scheduling problem based on the objective of maximizing the tugboat’s running time using different intelligent algorithms [9–11], and Wang et al. investigated the tugboat schedule ng problem considering a multi-service model with multiple waypoints, with the optimization objective of minimizing the cost, and solved it with an adaptive large-domain algorithm and applicable to large-scale problems [12]. This significantly promoted the study of Tug-SP models and represented a substantial improvement over traditional scheduling models. However, there are also limitations because, in practical port operations, tugboat efficiency is reflected by more than just maximum operating time. Port managers need to consider scheduling issues from various perspectives, including cost, efficiency, and environmental protection. A single objective function often fails to capture the complexity of real-world situations.

**Table 1:** Related vessel scheduling literature.

Authors	Focus	Optimization objective	Solution
wei et al. (2020) [6]	Tug-SP	Min, Total cost	BCA
Abou-Kasm et al. (2021) [7]	Tug-SP	Min, the maximum waiting time of a vessel.	BB
Jia et al. (2022) [8]	Tug-SP	Min, Ship delays, tugboat operating costs, and the number of ships that cannot be serviced	LR
Liu (2009) [9]	Tug-SP	Min, Maximum operating time of the tug	HESA
Xu et al. (2012) [10]	Tug-SP	Min, Maximum operating time of the tug	HSA
Kang et al. (2007) [11]	Tug-SP	Min, Maximum operating time of the tug	Ad-hoc algorithm
Wang et al. (2023) [12]	Tug-SP	Min, Total cost	ALNS
Goli et al. (2023) [13]	JSP	Min, makespan and total consumed energy	MOALO, MOKA, MOKSEA
Tirkolaee et al. (2023) [14]	MSW/LARP	Min,Total cost,Environmental discharge	MOSA-MOIWOA/GWO,PSO
Moharam et al. (2022) [16]	TL-SP	Min, Total penalty for delay and loss	DChOA
Zhong et al. (2022) [17]	Tug-SP	Min, Maximum completion time and total fuel consumption	NSGA-II

The second stage of the research takes into account other indicators reflecting the efficiency of the model, and the solution algorithms are gradually intelligent, but the applied algorithms have certain shortcomings in solving such problems as Tug-SP. Goli et al. studied the non-permutation flow-shop scheduling problem with the optimization objective of minimizing the completion time and the total energy consumed at the same time, and proposed Multi-objective Ant Lion Optimizer (MOALO), Multi-objective Keshtel Algorithm (MOKA) and Multi- objective Keshtel and Social Engineering Optimizer (MOKSEA) for solving the problem, and the experiments proved that these types of algorithms have good performance on the JSP [13]. Tirkolaee et al. jumped out of the JSP and used different heuristic algorithms to solve the Municipal Solid Waste (MSW) and Location Allocation Routing Problem (LARP) respectively, considering multiple metrics such as cost and time, to achieve better results [14,15]. Moharam et al. proposed a Discrete Chimpanzee Optimization Algorithm (DChOA) for the delay/loss (TL) penalty scheduling problem in discrete optimization problems, which is more advantageous than many other algorithms for solving the TL problem [16]. Zhong et al. establish a dual-objective tugboat scheduling model with maximum completion time and total fuel consumption as the optimization objectives, and choose NSGA-II algorithm for the solution, which achieves better results on the example of Guangzhou port [17].

Due to the current deficiencies in solving high-dimensional problems and inadequate convergence accuracy in tugboat scheduling algorithms, we have noticed a remarkable new intelligent algorithm in the field of scheduling in recent years—the Grey Wolf Optimization (GWO) algorithm proposed by Mirjalili, a scholar from Griffith University, Australia, in 2014 [18]. The advantages of this algorithm lie in its simple structure, few parameter settings, fast convergence speed, strong global optimization capability, and applicability to solving high-dimensional problems. As a result, GWO has been widely applied in various fields such as job-shop scheduling, engineering manufacturing, image information, and energy optimization [19–22].

However, GWO also has its limitations. It encounters conflicts when solving integer programming problems and is prone to getting stuck in local optima in the later stages of the algorithm. Many scholars have proposed improvements from various aspects such as control parameters, individual updates, and incorporating other strategies. Jordehi established an intelligent home energy management scheduling model and used GWO to solve it under the condition of integer encoding. By incorporating a penalty mechanism, the algorithm’s convergence accuracy was improved, to some extent, mitigating the conflicts in GWO when solving integer problems [23]. Liang et al. targeted the JSP and introduced a reverse learning strategy to increase population diversity. They combined it with simulated annealing to compensate for GWO’s tendency to fall into local optima, thereby enhancing the algorithm’s performance [24]. Zheng et al. proposed an optimal-worst reverse learning strategy for optimizing scheduling algorithms, enabling timely escape from local optima [25]. Furthermore, Zuo et al. built upon the optimal-worst reverse learning strategy and proposed a trend-optimizing reverse learning strategy for the Differential Evolution algorithm, further enhancing the algorithm’s ability to escape from local optima [26].

### 1.2. Objectives and contributions

This paper investigates the problem of harbor tugboat scheduling. Effective tugboat scheduling can save harbor cost and improve harbor navigation efficiency. A multi-objective tugboat scheduling model is constructed under taking into account tugboat operation time, port operation costs and port navigation efficiency. The improved Gray Wolf Optimization Algorithm is used to solve the problem, and as a result, the optimal scheduling plan is obtained, which provides effective decision-making reference and optimization direction for port managers.

The main contributions of this paper are as follows:

- This paper addresses the tugboat scheduling problem, which has been widely used in reality but has not received much attention.
- A new multi-objective tugboat scheduling model with optimization objectives that are more in line with realistic scenarios, including tugboat running time, tugboat fuel cost and tugboat power overflow, is proposed to better simulate the real situation.
- In order to solve the problem of integer coding conflicts and the tendency to fall into local optimization in GWO, the GWO algorithm is improved in this paper. This includes the introduction of a convergence parameter based on cosine mode, a dynamic weight adjustment mechanism with an inverse learning strategy for trend optimization to complement the current shortcomings of GWO.
- In the experimental part, the Taguchi method was tested to determine the optimal parameter combination of the algorithm. Simulation comparisons of the solution effects of IGWO are performed using multiple arithmetic cases of different scales by combining IGWO with CPLEX, Improved Particle Swarm Optimization (IPSO) [27], Discrete Chimp Optimization Algorithm (DChOA), Adaptive Large Neighborhood Search Algorithm (ALNS), GWO and IGWO are evaluated by comparing them and the results show that IGWO has better solution performance on TSP.

The rest of this paper is organized as follows:

Section 2 presents the tugboat scheduling model, including a detailed model description, parameter settings, and value ranges.

Section 3 introduces the improved Grey Wolf Optimization algorithm (IGWO), including the principles of GWO, the rationale and effects of the proposed improvements, and the process of applying IGWO to solve the Tug-SP problem.

Section 4 designs numerous simulation experiments and applies multiple algorithms to solve the problem, providing a comparative analysis to validate the performance of IGWO.

Section 5 is designed to further validate the effect and improvement direction of IGWO, to obtain the optimal scheduling plan to provide some reference value for port scheduling, and to design a sensitivity analysis scheme to consider the effect of cost on the tugboat scheduling model.

Section 6 is to optimize the tugboat scheduling plan for the integrated port area of Huanghua port, and to test the sensitivity of the unit usage cost of two types of tugboats in this port area, so as to provide further adjustment experience for the managers.

Section 7 sets out the conclusions and outlook.

## 2. Construction of port tug scheduling model

### 2.1. Problem description

**Fig 1:**
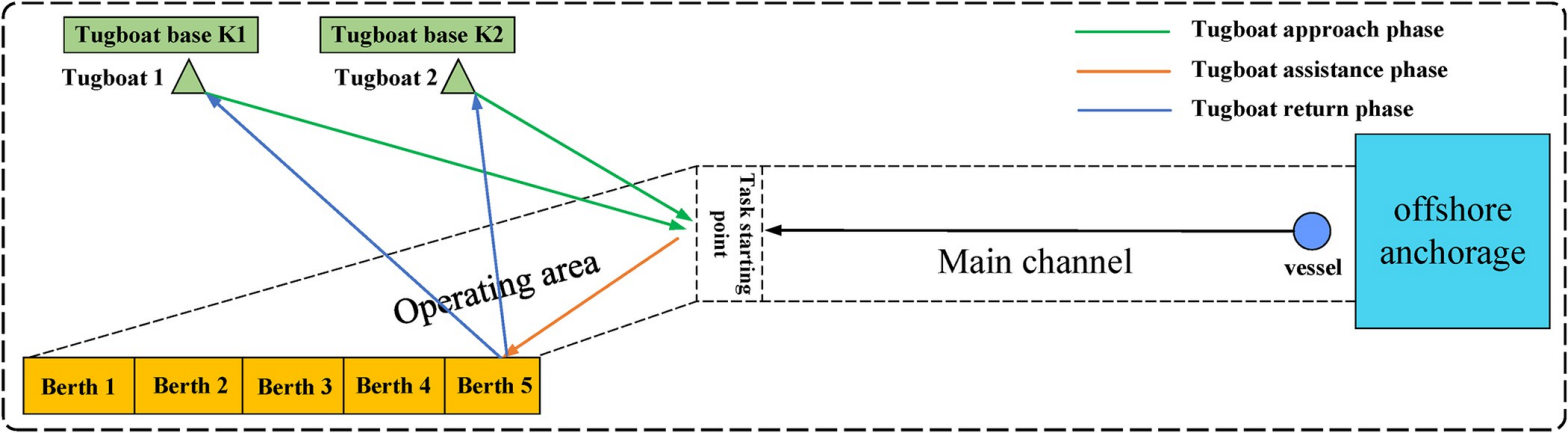
Example of two tugs assisting in berthing.

Tugboat berthing and unberthing tasks can be divided into three stages: the approach stage, the assist stage, and the return stage. The approach and return stages are considered as the process in which a tugboat operates without any load, while the assist stage is considered as the operational process. Taking the example of two tugboats assisting in berthing, the specific process is illustrated in Fig 1.

The assumptions for constructing the model are as follows:

1. Task scheduling: Prior to the start of tugboat operations, the port receives the daily vessel operational requirements and specific berthing/unberthing details. Tasks are assigned to each vessel in chronological order, taking into account the available number of tugboats, their power, and other relevant information. Tasks that are close in time (with similar start and end times) can be combined, utilizing shared tugboats.
2. Tugboat operations: After completing the assist stage of a task, the tugboat randomly returns to a base and waits for the next task (which may not be immediately following). Returning to the nearest base may not be advantageous for achieving the globally optimal solution.
3. Tugboat bases: Initially, the tugboats are randomly distributed among the tugboat bases. The time required for tugboats to enter or exit the bases is negligible.
4. Tugboat power: Each tugboat has a fixed power for propulsion. Tugboats with higher power have higher empty cruising speeds and fuel consumption. The cost of empty cruising per unit distance and the cost of assistance per unit time are also higher.
5. Navigation rules: The port has a one-way channel, and vessels cannot sail in parallel.
6. Task time windows: Based on the task scheduling information, the time interval for each vessel to complete the berthing/unberthing can be determined.

### 2.2. Parameter settings

The parameter settings for the tugboat scheduling problem, along with their descriptions, are presented in Table 2, while Table 1 is a two-tier table, where the first and third columns represent the parameter in the model, and the remaining two columns provide corresponding explanations. In addition, the individual tugboats in the model are meant to be similar tugboats and have different performance in terms of power, speed, and fuel consumption. In addition, the individual tugs in the model are meant to be similar tugs and have different performance in terms of power, speed, and fuel consumption. $L1_{ij}^{k}$ is related to the current position of the tugboat and the position of the target ship entering the harbor, and $L2_{ij}^{k}$ is related to the current position of the tugboat and the berth where the ship is docked.

**Table 2:** Parameters of the tugboat scheduling model.

Parameter type	Parameter	Description
Set	*I*	Set of tugboats, *i*∈*I* = {1,2,3,…,n}
	*J*	Set of tasks, *j*∈*J* = {1,2,3,…,m}
	*K*	Set of bases, *k*∈*K* = {1,2,3,…,l}
Input Parameters	$L1_{ij}^{k}$	Distance from the current position i of the tugboat to the start of the task j
	$L2_{ij}^{k}$	Distance from tug *i* at base k to the ending point of task *j*
	*Ct*_*i*_	Cost per unit distance traveled by tugboat *i* during transit
	*Co*_*i*_	the cost of unit travel time for tugboat *i* during the assistance operation
	*M*_*j*_	Minimum power requirement for task *j*
	*D*_*j*_	Tugboat demand for task *j*
	*P*_*i*_	Power of tugboat *i*
	$B_{i}^{k}$	Location of tugboat *i* at base *k* after completing the previous task
	*st*_*i*_	Time of arrival of vessel for task *j* at the starting point
	*et*_*j*_	Time of arrival of vessel for task *j* at the ending point
	*v*_*i*_	Empty cruising speed of tugboat *i*
Decision variables	*x*_*ij*_	0–1, 1 if tugboat *i* is assigned to task *j*, otherwise 0
	$y_{ji}^{k}$	0–1, 1 if tugboat *i* is at base *k* after completing task *j*, otherwise 0

### 2.3. Model establishment

Firstly, in order to improve the efficiency of port operations, it is necessary to minimize the operational time of tugboats during the berthing and unberthing process of vessels. The operational time of tugboats in the port refers to the total time spent on both idle operations and assisting operations for completing all tasks. The first objective function is shown in Eq (1).


$$F_{1}=\min\sum_{j}\left(\left(L1_{ij}^{k}+L2_{ij}^{k}\right)/v_{i}+et_{j}-st_{i}\right)$$ (1)

Additionally, the control of fuel costs for tugboats is considered. When tugboats assist vessels in berthing and unberthing operations, the main costs are based on power of tugboats and the duration of task execution. Therefore, in this study, the cost during the assistance phase is measured based on the assistance cost of different tugboats. The remaining phases involve unloaded travel and do not involve assistance phase, which can be measured by the cost per unit distance traveled by the tugboats. The cost of tugboat operations (during the assistance phase) is influenced by two factors. On the one hand, it depends on the power of the operating tugboat, with higher power resulting in higher costs. On the other hand, it is determined by the duration of the operation, which is governed by the berthing and unberthing schedule of the target vessel. The second objective function is shown in Eq (2).


$$F_{2}=\min\sum_{k}\sum_{i}\sum_{j}Ct_{i}(L1_{ij}^{k}+L2_{ij}^{k})y_{j-1,i}^{k}x_{ij}+\sum_{i}\sum_{j}Co_{i}(et_{j}-st_{i})x_{ij}$$ (2)

Furthermore, the overflow power of the tugboats is calculated. For tugboat-assisted berthing and unberthing tasks, tugboats should arrive within the time window for the target vessel to avoid delays in cargo handling and occupation of the designated berth, resulting in demurrage fees. Early arrival has less impact on the port, therefore, the waiting cost is not considered in this study. The third objective function is shown in Eq (3).


$$F_{3}=\min\sum_{i}\sum_{j}\left(P_{i}-M_{j}\right)x_{ij}$$ (3)

In summary, considering the optimization objectives of minimizing the maximum operational time of tugboats, fuel costs associated with idle and assisting operations, and the overflow power of tugboats, a new multi-objective tugboat scheduling model is established. The overall objective function of the model is shown in Eq (4).


$$MinF=min(F_{1},F_{2},F_{3})$$ (4)

$$\sum_{i}x_{ij}=D_{j},\forall j\in J;$$ (5)

$$y_{0i}^{k}=B_{i}^{k},\forall i\in I,k\in K;$$ (6)

$$P_{i}\geq M_{j}x_{ij},\forall i\in I,\forall j\in J;$$ (7)

$$\sum_{k}L1_{ij}^{k}y_{0,i}^{k}x_{i1}/v_{i}\leq st_{1},\forall i\in I;$$ (8)

$$et_{j-1}+\sum_{k}L1_{i,j-1}^{k}y_{j-1,i}^{k}x_{i,j-1}/v_{i}+\sum_{k}L2_{i,j-1}^{k}y_{j-1,i}^{k}x_{i,j-1}/v_{i}\leq st_{j},\forall i\in I,j\in \left\{2,3,\ldots ,NJ\right\};$$ (9)

$$y_{j-1,i}^{k}\geq x_{ij}\forall i\in I,\forall j\in J,\exists k\in K;$$ (10)

$$\sum_{k}y_{ji}^{k}\geq x_{ij},\forall i\in I,\forall j\in J;$$ (11)

$$\sum_{k}y_{ji}^{k}=1,\forall i\in I,\forall j\in J,k\in K;$$ (12)

$$y_{j-1,i}^{k}=y_{ji}^{k},\forall i\in I,\forall j\in J,k\in K:x_{ij}=0;$$ (13)

$$x_{ij},y_{ji}^{k}\in \{0,1\},\;\forall i\in I,\forall j\in J_{\circ }$$ (14)

The constraints of the model are represented by Eqs (5) to (14), Eq (5) represents the number of tugs allocated to each task. Eq (6) indicates the initial positions of the tugs before the first task. Eq (7) states that the tugs completing a task must meet the minimum power requirement for the current task. Eq (8) means that the tugs assigned to the first task should arrive at the starting point of the task before the start of the first task. Eq (9) denotes that after completing the previous task, the tugs should return to the docking base and reach the starting point of the current task in time. Eq (10) represents the requirement for the tugs to return to a specific base before proceeding to the next task. Eq (11) indicates that the number of tugs at a base should meet the required number of tugs to complete the tasks. Eq (12) states that a tug can only dock at one base at a given time. Eq (13) implies that in the absence of any tasks at the current stage, the tug remains at the previous base. Eq (14) signifies that the variables are integer values ranging from 0 to 1.

## 3. Improved gray wolf optimization algorithm

### 3.1. Principles of gray wolf algorithm

The GWO algorithm is characterized by simple structure, few parameters to be adjusted, easy to implement, etc., in which there are convergence factors that can be adjusted adaptively as well as the information feedback mechanism, which can achieve a balance between local optimization and global search, and GWO has a good performance in terms of solution accuracy and convergence speed.

The solution of multi-objective problems is often limited by the complexity of the solution space, while the global optimization ability and high-dimensional solution performance of GWO can compensate for this limitation to a certain extent, so that GWO has better robustness and applicability in multi-objective problems after parameter adjustment.

(1) Social hierarchy

**Fig 2:**
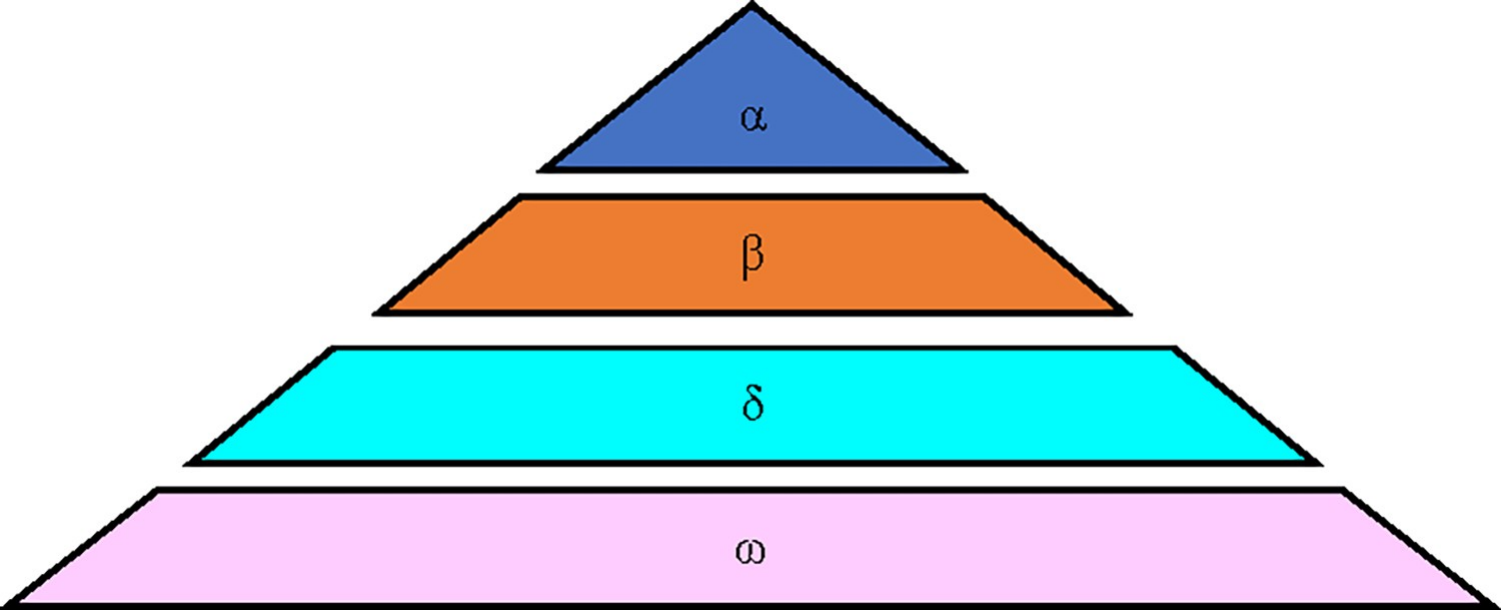
Grey Wolf group social hierarchy.

GWO is optimized by simulating the predation behavior of grey Wolf groups. Grey Wolf groups have a strict social hierarchy, as shown in Fig 2, which is divided into four classes. The specific meanings of each social hierarchy in the figure are as follows:

Level 1: Alpha wolf (α). The leader of the pack is responsible for leading the pack to hunt prey, which is the optimal solution in the optimization algorithm.

Level 2: Beta wolf (β). Assisting the alpha pack, the sub-optimal solution in the optimization algorithm.

Layer 3: Delta wolf (δ). Obey the orders of alpha and beta, responsible for reconnaissance, lookout, etc. The α and β of fitness differences will decrease to δ.

Level 4: Omega wolf (ω). They update their positions around α, β, or δ.

(2) Hunting process

GWO mimics the predatory characteristics of a gray wolf pack, with the goal of tracking, surrounding, chasing, and attacking prey to achieve optimal search. The hunting process of gray wolves is implemented based on Eqs (15) and (16):

$$\vec{D}=|\vec{C}•{\vec{X}}_{P}(t)-\vec{X}(t)|$$ (15)

$$\vec{X}(t+1)={\vec{X}}_{P}(t)-\vec{A}•\vec{D}$$ (16)

While *t* represents the current iteration count, ${\vec{X}}_{P}$ represents the position vector of the prey, $\vec{X}(t)$ represents the position vector of the current gray wolf; A and C are cooperative coefficient vectors that vary according to Eqs (17) and (18):

$$\vec{A}=2\vec{a}•{\vec{r}}_{1}-\vec{a}$$ (17)

$$\vec{C}=2{\vec{r}}_{2}$$ (18)

During the hunting process of the wolf pack, a linearly approaches 0 from 2; *r1* and *r2* are random vectors in the range [0, 1]. Clearly, as the iteration progresses, a gradually decreases to 0, causing the gray wolves to approach the prey. Simultaneously, *r1* and *r2* provide the opportunity for the wolves to escape from local optimal solutions.

### 3.2. Convergence parameters based on cosine variation

The magnitude of the cooperative coefficient vector *A* directly affects the overall and local optimization capabilities of the algorithm. Eq (17) indicates that *A* is controlled by the convergence parameter a, which linearly decreases from 2 to 0 with the number of iterations. However, during the operation and search of the algorithm, this linear variation does not fully reflect its optimization process. Hou et al. improved the gray wolf optimization algorithm by introducing a non-linear convergence parameter [28]. Based on this theory, this study introduces a convergence parameter expression based on cosine variation:

$$a=a_{final}+\left(a_{initial}-a_{final}\right)\frac{1+{\left[\cos((t-1)\pi /\left(t_{\max}-1\right))\right]}^{n}}{2},t\leq \frac{1}{2}t_{\max}$$ (19)

$$a=a_{final}+\left(a_{initial}-a_{final}\right)\frac{1+{\left|\cos((t-1)\pi /\left(t_{\max}-1\right))\right|}^{n}}{2},\frac{1}{2}t_{\max}\leq t\leq t_{\max}$$ (20)

**Fig 3:**
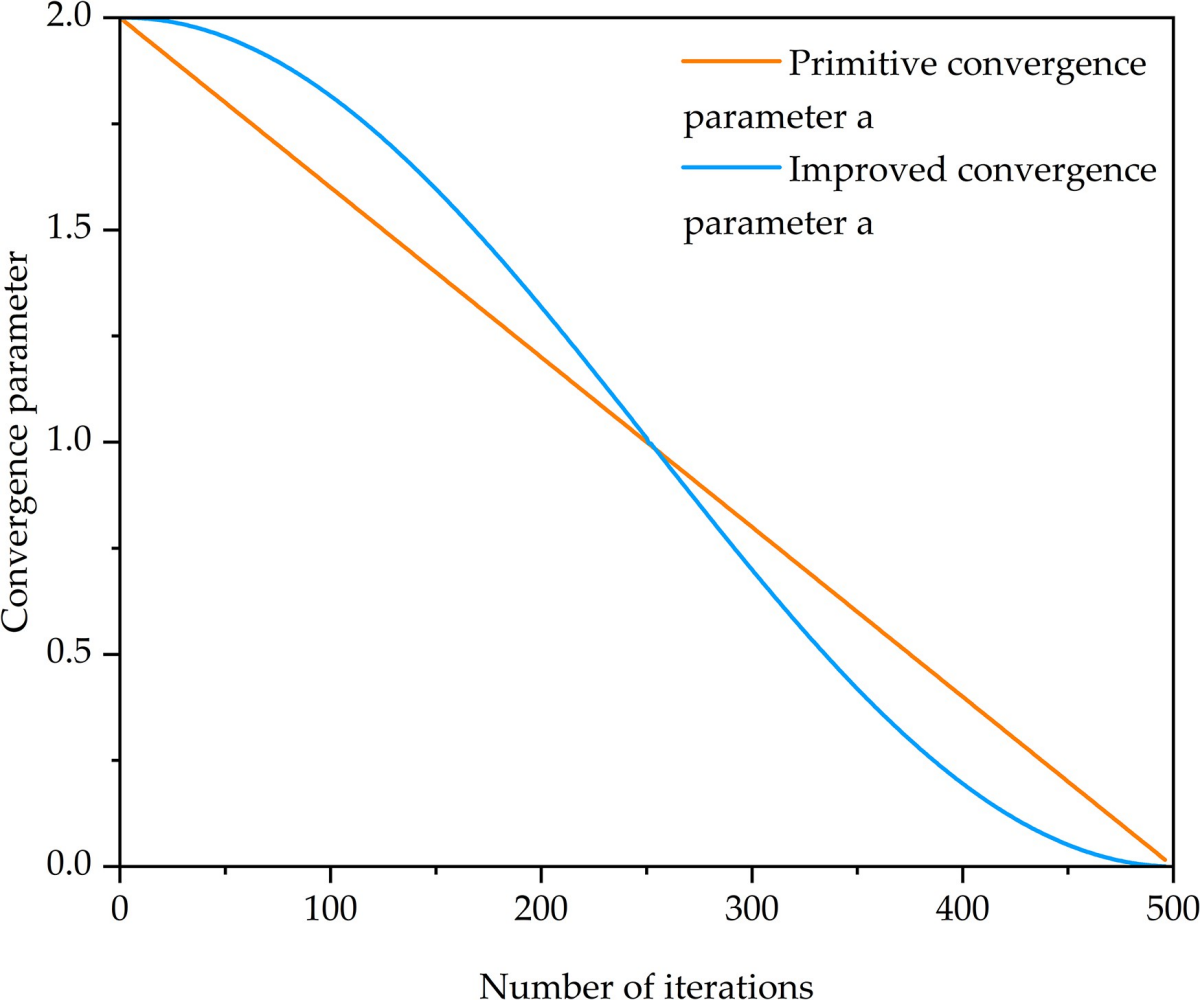
Convergence parameter variation illustration.

While *a*_*initial*_ and *a*_*final*_ respectively denote the initial and final values of the convergence parameter a, while n represents the decreasing index, 0≤*n*≤1. The convergence parameter varies over time as shown in Fig 3. From the change in the absolute value of the slope in the graph, it can be observed that the descent pattern of this convergence parameter is slow-fast-slow. This indicates that the improved convergence parameter decreases slowly in the initial stage, enabling a global search over a wide range. In the middle stage, it decreases rapidly, allowing faster encirclement of the prey and positioning in the vicinity of the optimal solution. In the final stage, it decreases slowly, enhancing the algorithm’s local search capability and improving algorithm accuracy.

### 3.3. Dynamic weight correction and update mechanism

In the original Grey Wolf Optimization (GWO) algorithm, each individual has the same learning degree towards the alpha, beta, and delta wolves, which hinders the ability of the new generation individuals to become more excellent through learning from the alpha wolf. To address this limitation, Meng et al. proposed a nonlinear dynamic learning weight mechanism [29]. however, it still couldn’t fully exploit the learning capability of individuals. In practical situations, the excellence of alpha, beta, and delta wolves is ranked as α>β>δ. Therefore, a dynamic weight mechanism based on step length Euclidean distance is introduced initially to dynamically adjust the learning weights according to the differences among the alpha, beta, and delta wolves. The specific formulas are shown in Eqs (18) and (19).


$$W_{1}=\frac{\left|{\vec{X}}_{1}\right|}{\left|{\vec{X}}_{1}|+|{\vec{X}}_{2}|+|{\vec{X}}_{3}\right|},W_{2}=\frac{\left|{\vec{X}}_{2}\right|}{\left|{\vec{X}}_{1}|+|{\vec{X}}_{2}|+|{\vec{X}}_{3}\right|},W_{3}=\frac{\left|{\vec{X}}_{3}\right|}{\left|{\vec{X}}_{1}|+|{\vec{X}}_{2}|+|{\vec{X}}_{3}\right|}$$ (21)

$$\vec{X}(t+1)={\vec{X}}_{1}•W_{1}+{\vec{X}}_{2}•W_{2}+{\vec{X}}_{3}•W_{3}$$ (22)

The tugboat scheduling problem is typically addressed using real-number encoding. However, the updating process in the Grey Wolf Optimization (GWO) algorithm often involves non-integer solutions. Moreover, the solution individuals in tugboat scheduling problem are subject to constraints, such as the non-repetition of tugboats for the same task and the minimum power requirement for each task. After the application of the GWO algorithm, the individuals may easily produce infeasible solutions. To address this issue, a cross-repair mechanism is proposed in combination with the aforementioned dynamic weight adjustment mechanism to optimize non-integer solutions. The specific steps of the dynamic weight correction and updating mechanism are as follows:

1. Based on the positions of the current wolf and the contemporary alpha, beta, and delta wolves in the population, as well as their respective cooperative coefficient vectors A and C.
2. Calculate the distance vectors between the current wolf and each of the alpha, beta, and delta wolves, and perform preliminary individual updates based on the dynamic weight mechanism using Formulas (18) and (19).
3. Correct the preliminary updated individuals to integer solutions (which are likely to still be infeasible solutions).
4. Obtain the set of eligible tugboats based on the minimum power requirement for each task.
5. Take the intersection between the infeasible integer solutions obtained in step 3 and the set of eligible tugboats from step 4, and select the individual that is closest to the infeasible integer solution to complete the update of the current individual. If there are missing parts in the individual, they are randomly selected from the remaining tugboats.
6. Perform the final update on all individuals in the Grey Wolf population following the above steps, thereby completing the overall update of the Grey Wolf population.

**Fig 4:**
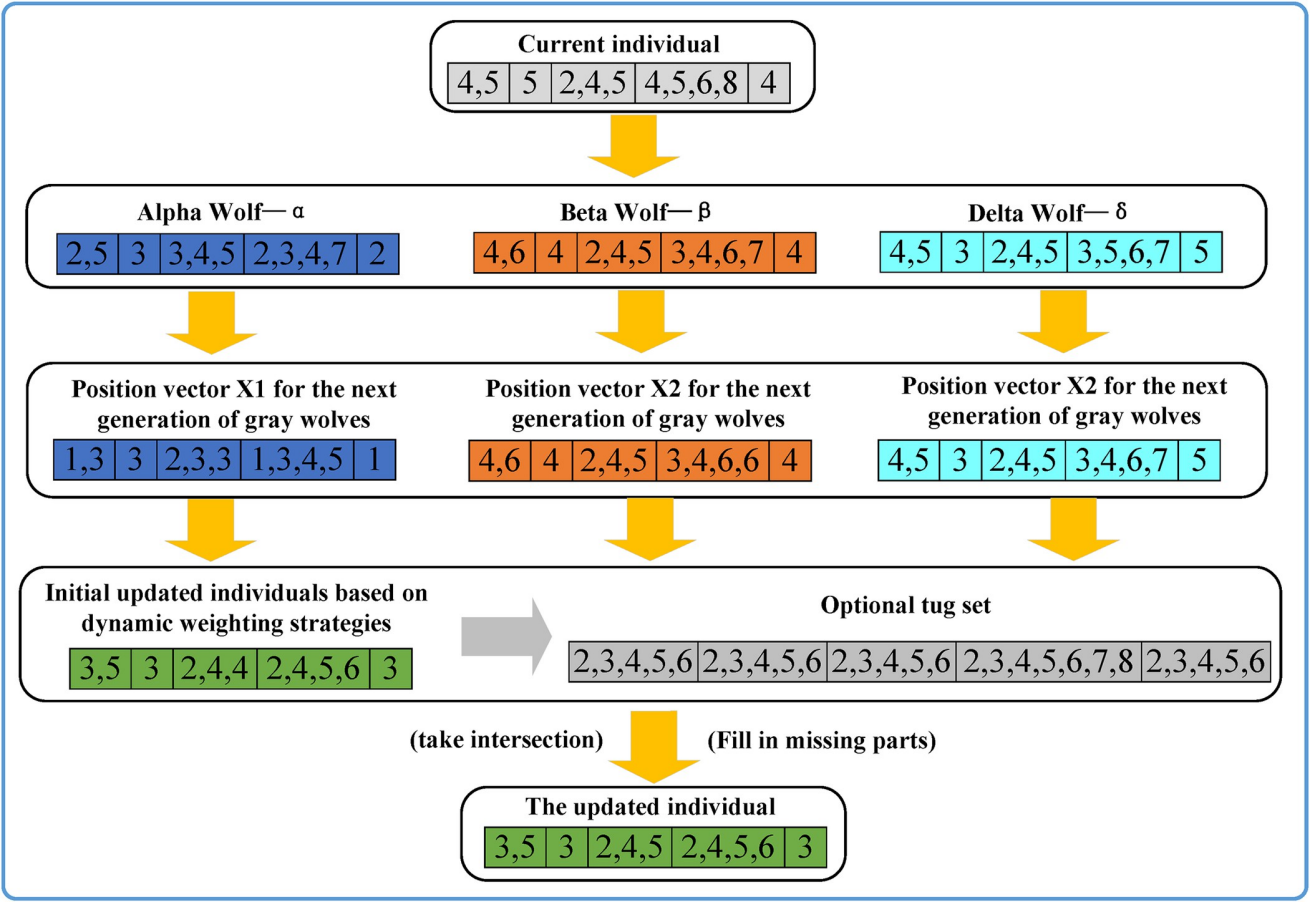
Dynamic Weight correction and updating example.

Taking the example of 5 tasks and 8 tugboats, Fig 4 demonstrates the process of cross-repair updating. The individual is composed of the tugboats required for all tasks. In this process, the current individual is cross-fused with the parameters A, C, and the contemporary alpha, beta, and delta wolves of the current generation. After rounding, the next generation’s Grey Wolf position vectors X1, X2, and X3 are obtained. The vectors are then centered and adjusted, and their intersection with the set of eligible tugboats is taken (with the remaining missing parts of the individual randomly selected). After multiple individual updates, the final result is a new generation of Grey Wolf individuals.

**Fig 5:**
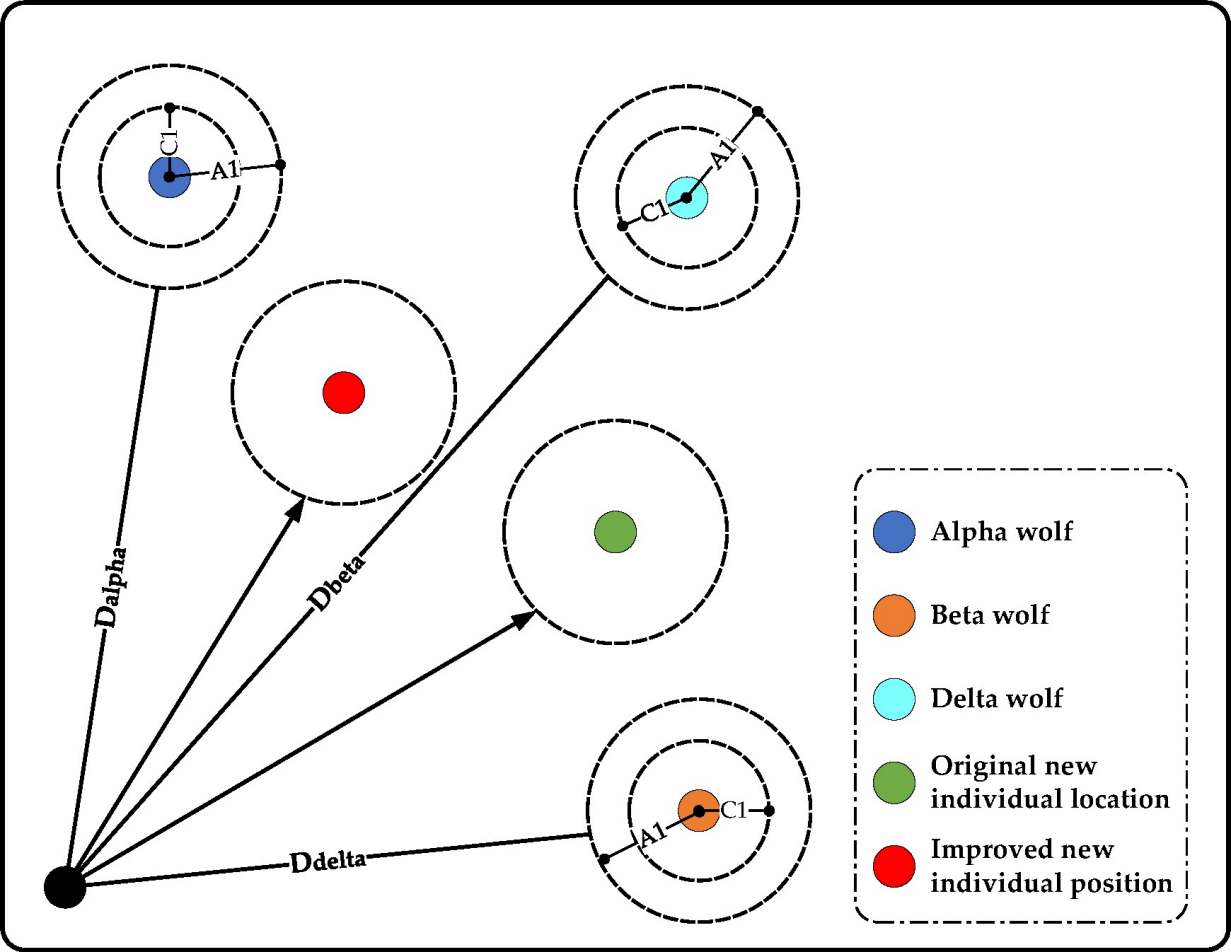
Comparison before and after dynamic weight update.

The application of the dynamic weight correction updating mechanism can make the new generation of individuals self-adaptive learning, and the learning rate of better wolves is higher. As shown in Fig 5, in the original grey Wolf algorithm, the position of the new individual is fixed in the center of the three grey Wolf position vectors X1, X2 and X3, which indicates that the new individual has the same learning degree to the three wolves. After applying the dynamic weight correction and update mechanism, the position of the new individual can be made closer to the better head Wolf, so this mechanism can enhance the convergence performance of the grey Wolf algorithm.

### 3.4. Towards excellent inverse learning strategy

The inverse learning strategy, introduced by Tizhoosh in 2005 [30], is a novel algorithm that enhances the global optimization capability of various algorithms, thereby improving their search efficiency. Building upon the theory of elite inverse learning, Li et al. incorporated a reverse learning mechanism for the best and worst individuals [31], as calculated in Eq (20).

where *x*_*ij*_ represents the current individual, *lb* represents the upper bound of the current individual, *ub* represents the lower bound of the current individual, and *x*_*r*_ represents the reverse solution.


$$x_{r}=lb+ub-x_{ij}$$ (23)

**Fig 6:**
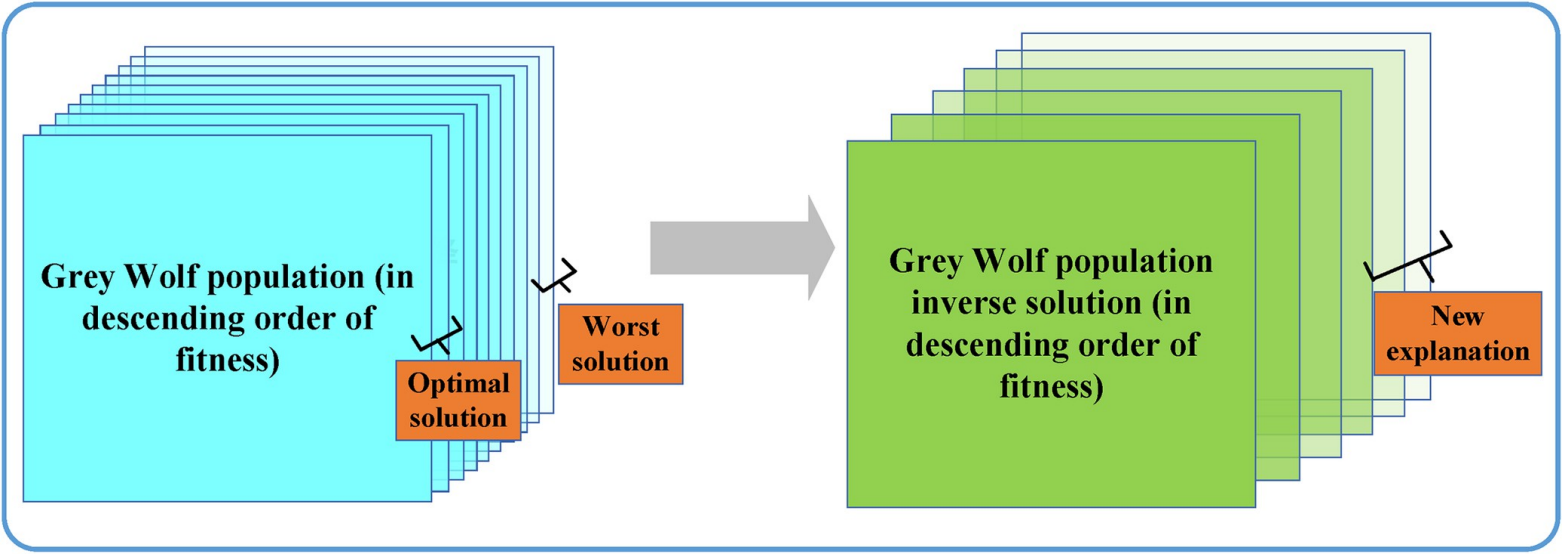
Towards excellent reverse learning strategy.

Ordinary elite inverse learning strategies only apply reverse learning to the best individuals in the population. However, the worst individuals in the population also contribute to diversity accumulation. Considering the characteristics of the Grey Wolf Optimization (GWO) algorithm, as shown in Fig 6, this paper selects the top 10% best and worst individuals from the wolf pack.

Their respective inverse solutions are calculated using Eq (23). These reverse solutions are then sorted based on their fitness values, and the lower half of the sorted individuals with the lowest fitness values are selected as new solutions. The algorithm randomly replaces individuals in the wolf pack with these new solutions, thereby enhancing the algorithm’s ability to escape local optima.

### 3.5. IGWO solving process

**Fig 7:**
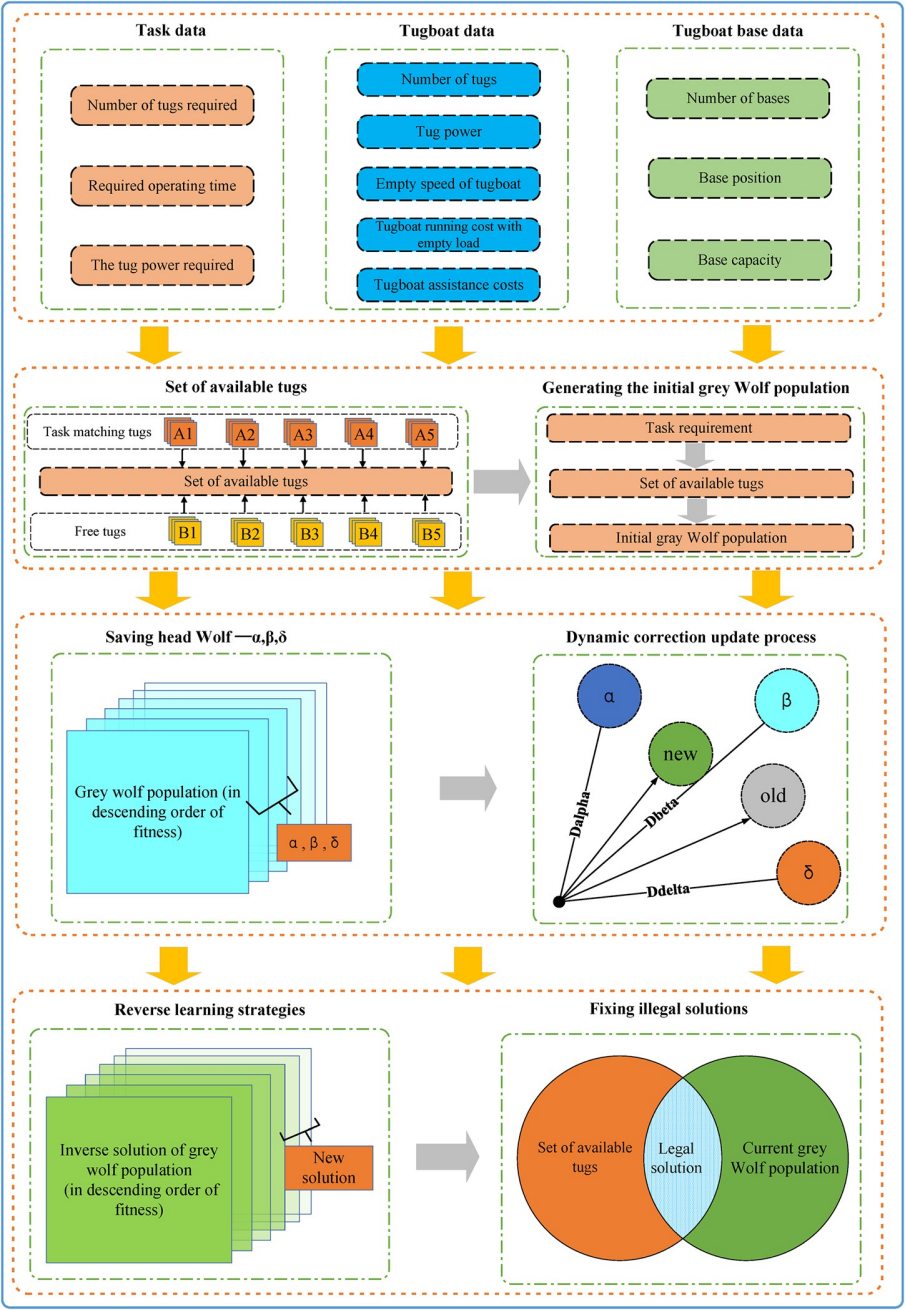
IGWO solution process.

Based on the above theoretical methods, this paper adopts the new IGWO algorithm to solve the tugboat scheduling model. The specific solution process is shown in Fig 7. The solution process is divided into four stages, which are as follows:

1. The first stage: First obtain the relevant information of the port task, and enter the task data, including the number of tugboats required for each task, the required working time, and the required tugboat power. The second is the data of the existing tugboats in the port, including the number of tugboats, power, idle speed, idle cost and navigational aid cost, two of which are jointly set by local regulations, the Maritime Authority and the port company. Finally, the data of the tugboat base, including the number and capacity of the tugboat base, as well as the location of the base.
2. The second stage: tasks will match a certain number and power of tugboats, which restricts the use of tugboats, and according to the ordering of tasks and the constraints of model conditions, the set of tugs currently available is obtained. Finally, according to the current available tugboat set, the initial grey Wolf population is randomly generated, which ensures that there will be no illegal solution in the initial solution.
3. The third stage: In the process of solving the gray Wolf algorithm, it is necessary to calculate the fitness of individual gray wolves in the gray Wolf population. The closer the individual is to the objective function, the greater the fitness is. All individuals are sorted according to the fitness, and the three gray Wolf individuals with the largest fitness are screened out, which are the first wolves of this generation α,β,δ. Next, all individuals need to learn from the modern leading Wolf to update themselves, and the updating method adopts the dynamic revision update mechanism, which has been explained in detail in the previous article.
4. The fourth stage: the update in the third stage satisfies the convergence process of the gray Wolf algorithm, but the algorithm is still prone to local optimization. Therefore, the optimal reverse learning strategy is adopted in the fourth stage to update the gray Wolf population twice. Although the optimal reverse learning strategy can help the algorithm jump out of the local optimal, it is prone to illegal solutions after updating, so the illegal solutions are corrected at last. The specific method is to take the intersection of the available tugboat set and the current gray wolf population to generate a legal new solution. If the length of the new solution is insufficient, it is randomly selected from the set of available tugboats to make up for it.

## 4. Tugboat scheduling case study simulation

To validate the effectiveness of the IGWO algorithm in solving the tugboat scheduling problem, simulation experiments are conducted to compare different instances and multiple intelligent algorithms. The code for each experiment is implemented using Python 3.7 (with the CPLEX library called through Python functions). The simulation runs on a laptop with an 11th Gen Intel(R) Core(TM) i5-11400H @ 2.70GHz processor, 16GB RAM, and Windows (64-bit operating system).

### 4.1. Experimental parameter settings

To assess the performance of IGWO in harbor tugboat scheduling, we generate tugboat scheduling cases based on different scales. The scale of each instance is controlled by the number of tasks and tugboats. In addition to the parameters controlling the scale mentioned in Table 2, there are several common parameter ranges for all cases. These ranges are used to define constraint conditions and calculate various cost expenditures during the scheduling process. The parameter ranges are determined by referring to relevant data on China’s port charging standards [32], taking into account the specific model proposed in this study and the actual conditions of the one-way channel in the harbor. The parameter ranges are shown in Table 3. Table 3 shows the ranges of some parameters. Columns 1 and 3 are the parameters, columns 2 and 4 indicate the parameter ranges, and columns 3 and 6 denote the units of the parameters. The explanations for the parameters have been given earlier in the text and will not be repeated here.

**Table 3:** Parameter ranges in the test cases.

Parameters	Ranges	Units	Parameters	Ranges	Units
$L1_{ij}^{k}$	uniform(2,18)	n mile	*D*_*j*_	randint(1,5)	—
$L2_{ij}^{k}$	uniform(2,18)	n mile	*P*_*i*_	randint(4000,6000)	kw
*Ct*_*i*_	ranint(400,600)	元/n mile	*M*_*j*_	randint(4000,5000)	kw
*Co*_*i*_	ranint(150,250)	元/min	*v*_*i*_	uniform (8,16)	n mile/h

### 4.2. Feasible solution setup

Given the numerous constraints in the model, it is necessary to set up the generation of feasible solutions. The constraints to be considered can be divided into three aspects: the demand of each task for tugboats, the operational limitations of the tugboats themselves, and the capacity limitations of the tugboat base.

The setting of feasible solutions for the multi-objective tugboat scheduling model is shown in Algorithm 1, which first obtains the task ship and tugboat data through the port, and generates the set of available tugboats through this data to clarify the number of tugboats required for each task. On this basis, a single feasible solution is generated, and the population of feasible solutions is generated according to the population size parameter *N*_*P*_.

**Algorithm 1:** setup of feasible solutions

1: **Input:** number of tugboats needed for each task Dj, minimum tugboat power for each task Mj, power of each tugboat Pi, number of feasible solutions Np, number of tasks task_num, number of tugboats tugboat_num

2: **output:** population of feasible solutions pop_solution

3: choose_tugboat = create empty list ()  // create set of available tugboats

4: **for** j ← 0 to ( task_num - 1 ) **do**   // j denotes the task number

5:  **for** i ← 0 to ( task_num - 1 ) **do**  // i denotes the tugboat number

6:    **If** the current tugboat’s power meets the mission minimum then

7:        add the current tugboat number i to choose_tugboat

8:    **end if**

9:  **end for**

10: **end for**

11: function single_s(Dj, Pi, choose_tugboat)   // generate a single feasible solution

12:  solution = = create empty list ()   // Create individual feasible solutions

13:  **for** j ← 0 to ( task_num - 1 ) **do**   // j denotes the task number

14:      ran**do**mly select Dj from choose_tugboat[ j ] and add it to solution.

15:   **end for**

16:  pop_solution = create empty list

17: pop_solution = create empty list () // create set of populations of feasible solutions

18: **for** k ← 0 to (Np - 1) **do**

19:  solution = single_s(Dj, Pi, choose_tugboat)

20:  add solution to pop_solution

21: **end for**

### 4.3. Algorithm parameter optimization

The parameters that can be adjusted in the IGWO algorithm include: the population size *N*_*P*_, the number of iterations *N*_*t*_, and the convergence parameter a, in which the convergence parameter a has been adjusted based on the cosine law, so the next two parameters can be adjusted.

Different combinations of algorithm parameters will affect the solution performance of the algorithm, so the Taguchi method is used to test to determine the best combination of parameters. One of the two parameters, three levels of parameter combination experiments are shown in Table 4.

**Table 4:** Algorithm parameter levels.

level	*N*_*P*_	*N*_*t*_
1	100	500
2	200	1000
3	300	1500

Next, the results of each parameter combination were tested at three different scales, respectively, and the scale of the test cases is shown in Table 5. The test results were measured using the signal-to-noise ratio (*SNR*), which is calculated as shown in Eq (24):

$$SNR=-30\log(\frac{1}{30}\sum_{i=1}^{10}\sum_{i=1}^{3}\frac{1}{y_{ij}^{2}})$$ (24)

**Table 5:** Parametric test arithmetic.

Scale	*J*	*I*
1	10	15
2	35	40
3	70	75

Where *y*_*ij*_ represents the test results of each group.

Orthogonal experiments were carried out according to three levels of each parameter, and each group was tested 10 times, and the test results are shown in Table 6, and it can be seen by observing the table that the SNR value reaches the minimum in the 5th group of experiments, so this paper sets the algorithm parameters as the number of populations *N*_*P*_ = 200, and the number of iterations *N*_*t*_ = 1000.

**Table 6:** Results of orthogonal experiments.

Experiment number	*N*_*P*_	*N*_*t*_	*SNR*
1	100	500	745.23
2	100	1000	744.35
3	100	1500	745.32
4	200	500	745.36
5	200	1000	743.70
6	200	1500	745.02
7	300	500	743.88
8	300	1000	744.23
9	300	1500	744.47

### 4.4. Small-scale simulation experiments

To verify the applicability of the IGWO algorithm in small-scale tugboat scheduling problems, 15 sets of different small-scale cases were designed for solving using the IGWO algorithm.

A comparison experiment was conducted with CPLEX, a mathematical solver, as shown in Table 7. The first column of the table represents the 15 sets of generated instances. The second column indicates the number of tasks in each instance, which is slightly larger than the number of target vessels since each task requires 1–5 tugboats. On average, the number of target vessels is 2.5 times the number of tasks. The third column represents the total number of available tugboats for each instance. The fourth and fifth columns show the results of CPLEX and IGWO, respectively, for each instance. The last column presents the deviation between the two solutions.

**Table 7:** Results of benchmark instances with CPLEX.

Instance	|*J*|	|*I*|	Bes.		Dev.
			CPLEX	IGWO	
I-1	4	4	32332.59	32332.59	0.00%
I-2	4	6	24079.15	24079.15	0.00%
I-3	4	8	19167.04	19167.04	0.00%
I-4	8	8	36153.51	36153.51	0.00%
I-5	8	10	34256.51	34256.51	0.00%
I-6	8	12	31164.84	31164.84	0.00%
I-7	12	12	50375.60	50572.38	0.39%
I-8	12	14	49093.31	49098.05	0.01%
I-9	12	16	48613.15	48725.42	0.23%
I-10	16	16	69679.33	70290.86	0.88%
I-11	16	18	65463.27	65813.09	0.53%
I-12	16	20	66939.73	67226.95	0.43%
I-13	20	20	NFL	78351.99	-
I-14	20	22	NFL	76493.90	-
I-15	20	24	NFL	74730.92	-

Among all the small-scale cases, IGWO achieved the same optimal value as CPLEX for the first 6 instances, with a deviation of 0.00%. For instances 7–12, the solutions of both methods were very close, with a deviation controlled within 0.9%. In the last 3 instances, CPLEX encountered a memory overflow issue and failed to obtain a solution, while IGWO’s solving process remained unaffected. At present, most of the ports in reality are small in scale, and it can be seen from the above examples that IGWO has good solving performance in the problem of small-scale tugboat scheduling. Therefore, the IGWO proposed in this paper has important practical significance.

### 4.5. Medium to large-scale simulation experiments

Similarly, we need to go to observe the performance of IGWO in medium and large-scale instances, because CPLEX cannot solve larger-scale problems, so the other three improved intelligent algorithms as well as the original Gray Wolf optimization algorithm were selected for experiments, designed 10 groups of medium and large-scale instances, and analyzed the strengths and weaknesses of the algorithms based on a number of indicators, as shown in Table 8.

**Table 8:** Comparative experiments of multiple intelligent algorithms.

Instance	|*J*|	|*I*|	Index	IPSO	DChOA	ALNS	GWO	IGWO	Gap.
1	25	30	Bes.	119002.61	93623.92	94048.71	94504.93	93447.34	6.83%
			Avg.	142026.59	111417.33	105139.57	107140.76	103927.73	
			Wor.	152764.06	124486.93	112901.51	116148.64	109036.61	
			Time.	180s	82s	78s	102s	62s	
2	30	36	Bes.	152692.71	121255.55	117544.02	122257.85	115477.87	10.09%
			Avg.	174200.47	141005.69	143502.04	144629.26	142908.41	
			Wor.	186298.94	153326.06	159233.21	155975.73	161391.94	
			Time.	250s	189s	231s	217s	152s	
3	35	42	Bes.	192478.95	162860.73	160767.40	153810.63	150024.40	10.42%
			Avg.	212380.20	176773.83	175431.02	176948.31	172579.45	
			Wor.	224419.35	192565.88	191482.37	192953.38	190539.97	
			Time.	317s	228s	302s	252s	213s	
4	40	48	Bes.	230108.77	199374.40	182611.38	186033.62	179437.75	10.07%
			Avg.	263869.37	213300.63	210490.88	212017.73	208496.66	
			Wor.	277939.72	231747.05	229070.88	230562.81	227671.51	
			Time.	500s	401s	563s	409s	387s	
5	45	54	Bes.	229393.18	189581.62	185708.69	188909.19	171987.06	13.31%
			Avg.	259643.34	212182.29	204473.59	208370.30	202554.32	
			Wor.	272414.85	232924.74	211276.00	219093.13	207030.39	
			Time.	706s	550s	872s	573s	539s	
6	50	60	Bes.	249042.09	247526.16	239323.85	240534.69	237852.64	2.56%
			Avg.	272463.42	279752.15	276611.51	271586.25	285825.07	
			Wor.	285047.03	301681.32	298816.60	291928.35	307450.13	
			Time.	905s	759s	1090s	817s	724s	
7	55	66	Bes.	275583.39	277146.11	264115.44	269248.53	260923.12	3.90%
			Avg.	304534.86	310437.80	311123.67	305463.26	313748.35	
			Wor.	321038.88	326728.91	331900.75	326776.65	341698.77	
			Time.	1387s	1088s	1492s	1112s	986s	
8	60	72	Bes.	316948.44	320466.96	306722.42	310673.79	303180.28	3.35%
			Avg.	298664.17	336509.45	292353.48	293695.23	290470.36	
			Wor.	317785.47	359640.02	308630.81	307779.61	307511.57	
			Time.	1601s	1378s	1729s	1497s	1263s	
9	70	82	Bes.	339533.31	349279.55	344390.80	345998.39	337861.13	2.01%
			Avg.	368751.88	387109.86	387773.44	390459.01	385848.77	
			Wor.	387197.29	411442.95	416347.52	419289.35	415051.57	
			Time.	1900s	1574s	2140s	1771s	1475s	
10	80	96	Bes.	384979.02	391798.27	377174.98	380064.96	371447.27	3.14%
			Avg.	387440.64	432322.66	390282.83	381957.47	361077.68	
			Wor.	406644.36	460047.22	394160.45	401146.07	388181.56	
			Time.	2367s	1924s	2648s	2193s	1802s	

Table 8 consists of the following columns: Instance Number, Task Quantity, Tugboat Total, and Solution Metrics including best value (Bes.), average value (Avg), worst value (Wor.) and solution time (Time.). Columns 5 to 8 represent the solution results of each intelligent algorithm for the given model, and column 9 indicates the gap in the optimal value (Gap.) between IGWO and other algorithms. Gap. is calculated using Formula (25), where a larger Gap. indicates the superiority of IGWO over other algorithms.


$$Gap.=1-3B{\mathrm{es}}_{4}/\sum_{i=1}^{3}Bes_{i}$$ (25)

Comparing the simulation results of different algorithms at different scales, it can be seen that the IPSO algorithm enables the algorithm to complete the fine search at a later stage through the nonlinear inertia weighting strategy, but it is limited by the algorithm’s own problem that the global optimal object of individual learning is only a single individual, which is not conducive to searching in the high-dimensional solution space. For the DChOA algorithm, although the algorithm improves the parameters and operators, the problem solving effect is not ideal for high-dimensional solution space, it can be seen that as the size of the solution increases, the dimensionality of the solution space is also increases, and the solving accuracy of DChOA highlights the lack of precision, and it is more likely to produce the phenomenon of premature maturity. The ALNS algorithm, due to the improved domain search operator, has a better performance compared to the previous algorithms, but the convergence speed is slower, and the GWO algorithm can easily fall into the local optimum without adjusting the convergence parameter alpha and the learning strategy, resulting in a lower solution accuracy. For the IGWO algorithm, observation of the experimental results shows that in examples 2–5, due to the relatively small size of the instances, the solution accuracy of IGWO is 10%-14% ahead of the other algorithms, and the remaining 7 instances still maintain a 2%-10% lead. In addition, the solution time of IGWO is significantly shorter than other algorithms, and its solution speed dominates at all scales.

This indicates that IGWO is more stable and outperforms all other compared algorithms, including the original GWO algorithm, in terms of overall performance. This is due to the tuning of the convergence parameters and the dynamic learning strategy, the better balance between the algorithm’s global and local convergence capabilities, and the introduction of the inverse learning strategy, which enables the algorithm to jump out of the local optimum in time.

## 5. Optimal solution tracking

Section 3 verified IGWO’s solution accuracy and stability was accomplished. In order to further enhance the algorithm’s performance and apply it to practical port operations, this Section presents a redesigned set of four instances with a large span of scales. The algorithm parameters remain the same as in Section 3. The aim is to track the optimal values and corresponding scheduling solutions of each intelligent algorithm, obtain their iteration curves, analyze the directions for improving IGWO, and ultimately provide a reference for practical port operations based on the optimal scheduling solution obtained through IGWO.

### 5.1. Validation of optimal solutions

**Fig 8:**
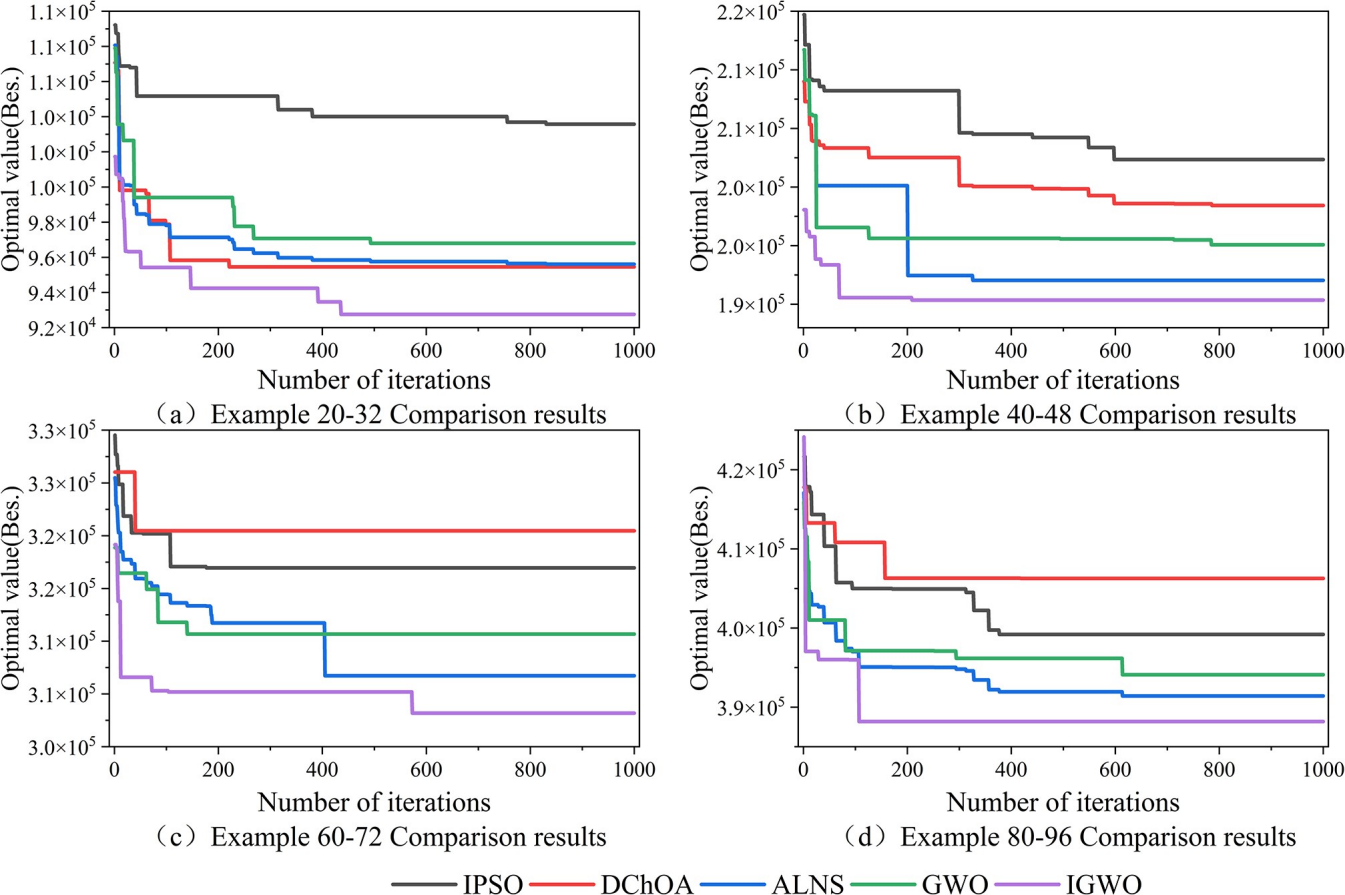
Iteration curves of each algorithm.

As shown in Fig 8, while (a), (b), (c), and (d) correspond to instances with task-tractor ratios of 20–32, 40–48, 60–72, and 80–96 respectively. According to the iteration curve, it can be observed that both HNGA and GWO algorithms suffer from the disadvantage of easily converging to local optima. Although IPSO improves the inertia weight, its performance is poor for moderate-scale problems.

The IGWO algorithm outperforms other comparative algorithms in terms of solution accuracy but has some limitations. Further improvements could focus on enhancing its ability to escape local optima in the later stages of the algorithm.

### 5.2. Optimal scheduling solution

**Fig 9:**
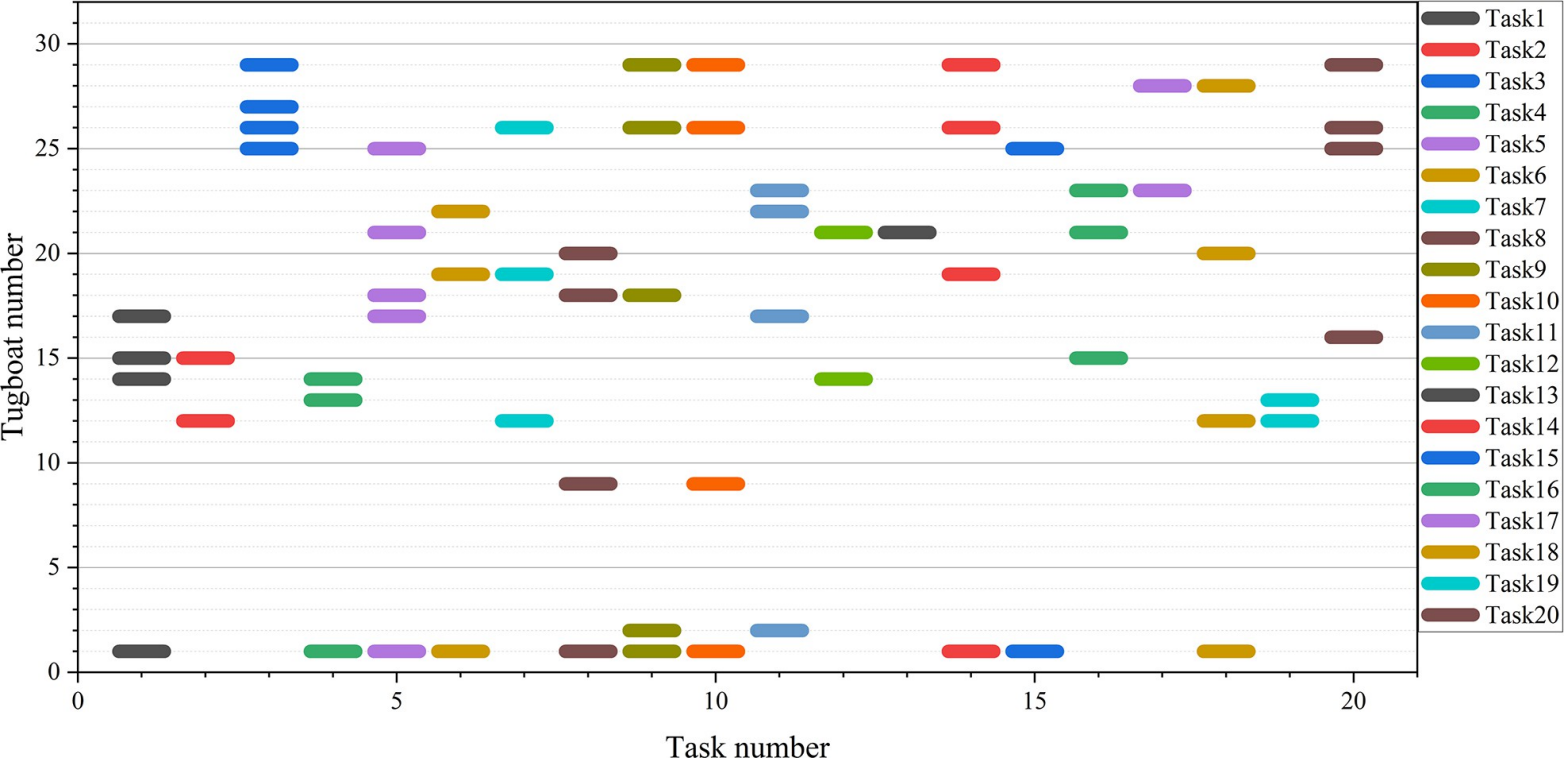
Gantt chart of optimal scheduling scheme for case 20–32.

To apply this algorithm to practical port operations, case (a) was selected as an example. The Gantt chart in Fig 9 represents the optimal scheduling plan generated by IGWO, providing a clear view of the assigned tugboat numbers and the sequence in which they perform tasks. It serves as a reference for tugboat scheduling in port operations. For example, tugboats assigned to serve Task 1 are Tugboat 1, Tugboat 14, Tugboat 15, and Tugboat 17. The sequence of tasks performed by Tugboat 12 is Task 2—Task 7—Task 18—Task 19. Additionally, it can be observed that Tugboat 1 is frequently assigned tasks, while Tugboats 3–8 remain idle. This is related to the existing tugboat capacities and task requirements. To address this, further optimization can be achieved by introducing constraints on continuous tugboat operations in the model.

### 5.3. Sensitivity analysis

This section focuses on the sensitivity of the objective function to the tugboat’s unloaded travel cost and pilotage cost. The optimal value (Bes.) is used as the evaluation criterion. The simulation experiments in this chapter continue to utilize the four instances introduced in Section 4.

**Fig 10:**
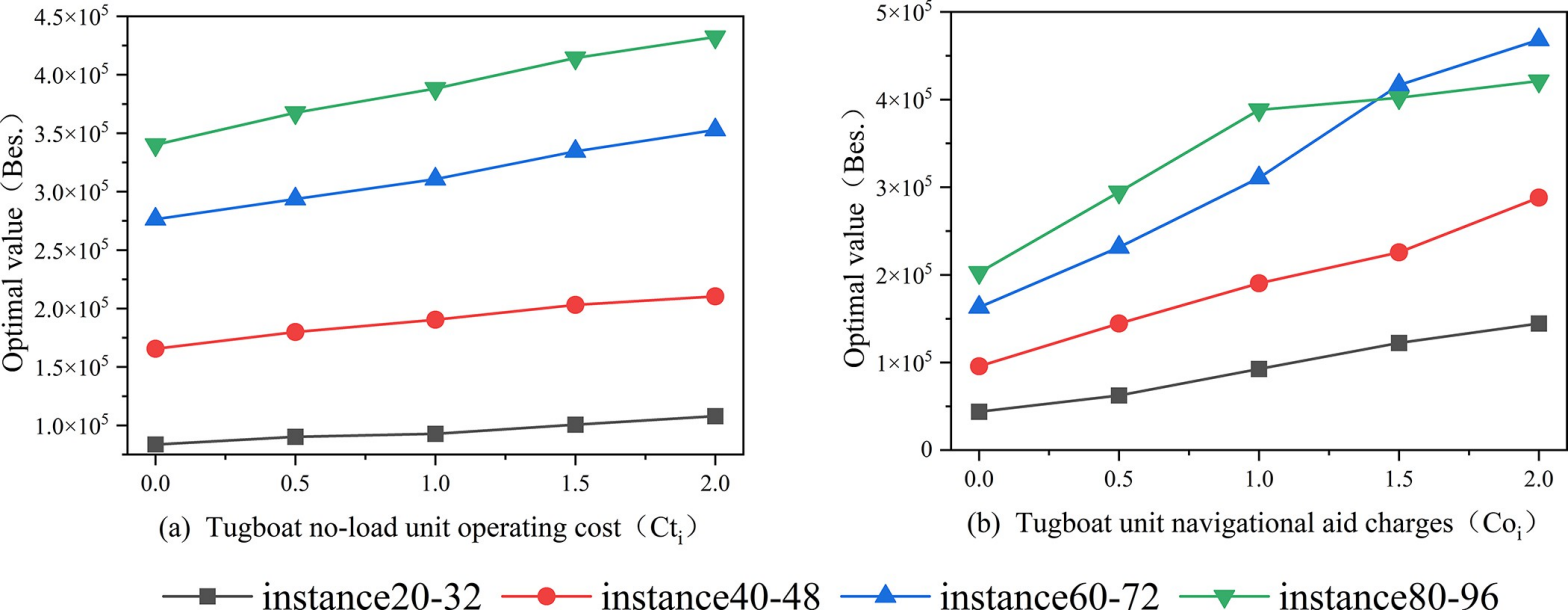
Sensitivity analysis of tugboat scheduling.

Firstly, a sensitivity coefficient is defined with a range of [0, 2] and a step size of 50%. It is used to test the impact of tugboat’s unloaded travel cost (*Ct*_*i*_) and navigation assistance cost (*Co*_*i*_) on each set of cases. The sensitivity performance of *Ct*_*i*_ and *Co*_*i*_ in the four sets of cases is shown in Fig 10, while (a) shows the curve of the optimal value with respect to the sensitivity coefficient of idle cost, while panel (b) depicts the curve of the optimal value with respect to the sensitivity coefficient of navigation assistance cost.

From Fig 10 it can be seen that sensitivity is directly proportional to the slope of the curve. Analyzing Fig 10-(A), it can be observed that in small-scale instances, changes in *Ct*_*i*_ do not significantly affect the variation of the optimal value, only exerting a relatively larger impact on the optimal value in larger-scale cases. Conversely, in Fig 10-(B), *Co*_*i*_ has caused significant disturbances to the optimal value across instances of various scales.

In conclusion, the tugboat scheduling model established in this paper is significantly influenced by the tugboat assistance cost, and it is particularly affected by the tugboat unloaded travel cost in large-scale scenarios. This provides some recommendations for port managers. They should strive to minimize the tugboat assistance cost while also considering the unloaded travel cost, especially in large-scale ports, in order to achieve cost control and operational efficiency.

## 6. Huanghua port example study

In order to validate the effectiveness of the algorithm and the model, an example study is conducted with the integrated harbor area of Huanghua Port in China, which is about 9.5 nautical miles in length and is now in operation with 11 berths, 1 tugboat base, and 2 mission start points.

The data of May 1, 2022 is selected for the study of this port area, and the information of the tugboat configuration of this port area and the incoming ship data of that day are shown in S2 Table, the tugboat scheduling of that day in this port area is optimized by using the IGWO algorithm, and the parameters of the algorithm are the same as in the previous section, which results in the optimal operation scheme as shown in Table 9. The 21 vessels on that day are divided into 8 tasks, and the optimal order of tugboats to perform each task can be clearly seen through this table.

**Table 9:** Tugboat optimum operating program.

Number of Task	Number of Vessel
1	4,5
2	1,2,5
3	1,4,5,6
4	5
5	7
6	1,8
7	2
8	4,5,6

In addition, the same method was used to test the sensitivity of tugboat idling cost *Ct*_*i*_ and aids to navigation cost *Co*_*i*_ to the optimal value Bes. as shown in Table 10, where the first column of the sensitivity coefficients indicates the proportion of the change in the two factors, and the second and third columns indicate the extent to which the change in *Ct*_*i*_ and *Co*_*i*_, respectively, affects the optimal value (Bes.). In this example, the effect of the change in *Ct*_*i*_ is small, while the effect of *Co*_*i*_ is large. Therefore, if conditions permit, port managers should try to reduce the cost of tugboat navigation assistance *Co*_*i*_, which can more efficiently reduce the cost of port operation as well as improve the efficiency of port navigation.

**Table 10:** Sensitivity analysis of Huanghua Port.

Sensitivity factor	Variation of Bes.(*Ct*_*i*_)	Variation of Bes.(*Co*_*i*_)
0	97.82%	75.67%
0.5	98.91%	87.87%
1	100.00%	100.00%
1.5	101.15%	112.16%
2	101.96%	124.46%

## 7. Conclusion and future work

This paper proposes a novel tugboat scheduling model based on different power levels of tugboats and vessels. Multiple objective functions are established, including in-port operation time, various tugboat costs, and overflow power. The study explores the application of the Improved Grey Wolf Optimization (IGWO) algorithm to the tugboat scheduling problem. The algorithm’s performance is verified through simulation experiments at various scales. The results show that IGWO optimizes several other comparative algorithms, and finally the research in this paper is applied to the integrated port area of Huanghua Port to obtain the optimal scheduling scheme with effective management insights.

The proposed tugboat scheduling model in this study simulates the real-world scenario of tugboats assisting ships in entering and leaving ports. Through IGWO optimization, it generates efficient tugboat scheduling schemes that lead to fuel and manpower savings for ports while also reducing carbon emissions to some extent. This offers new insights and methods for promoting the sustainable development of maritime transportation.

This paper establishes a new tugboat scheduling model based on the task scheduling theory, but the model and algorithm can be further optimized in the future, model-wise the objective function can be replaced according to the actual needs of the port and the constraints of continuous operation of tugboats and inter-port scheduling can be added. In terms of algorithms, deep reinforcement learning is noteworthy, but such algorithms have high environmental requirements, and whether they are applicable to the tugboat scheduling problem needs to be studied.

## Supporting information

S1 Appendix Port billing schemes. The S1 Appendix describes the rules and regulations related to port billing in China.
(DOCX)

S1 Table Tugboat scheduling simulation dataset. The S1 Table shows the base data for the simulation example of this study.
(XLSX)

S2 Table Data of Huanghua Port Comprehensive Port Area. The S2 Table contains three sub-tables representing the basic data of the port area, the ships on a particular day, and the pre-processed ship data.
(XLSX)

## References

[1] Lin H N, Zeng W J, Luo J, Nan G F. An analysis of port congestion alleviation strategy based on system dynamics[J]. Ocean & Coastal Management, 2022, 229:106336. doi: 10.1016/j.ocecoaman.%202022.106336 36059572 PMC9417887

[2] Singh S, Dwivedi A, Pratap S. Sustainable Maritime Freight Transportation: Current Status and Future Directions[J]. Sustainability, 2023, 15(8). doi: 10.3390/su15086996

[3] Wu Z Z, Wang S Z, Yuan Q M, Lou N Y, Qiu S Y, Bo L, et al. Application of a deep learning-based discrete weather data continuousization model in ship route optimization[J] Ocean Engineering,2023, 285(2): 115435. doi: 10.1016/j.oceaneng.2023.115435

[4] Xiu G Y, Zhao Z X. Sustainable Development of Port Economy Based on Intelligent System Dynamics[J]. IEEE Access, 2021,9: 14070–14077. doi: 10.1016/S1876-3804(11)60009-8

[5] Petris M, Pellegrini P, Pesenti R. Models and algorithms for an integrated vessel scheduling and tug assignment problem within a canal harbor[J]. European Journal of Operational Research, 2022, 300(3): 1120–1135. doi: 10.1016/j.ejor.2021.10.037

[6] Wei X Y, Jia S, Meng Q, Tan K C. Tugboat scheduling for container ports[J]. Transportation Research Part E-Logistics and Transportation Review, 2020,142. doi: 10.1016/j.tre.2020.102071

[7] Abou-Kasm O, Diabat A, Bierlaire M. Vessel scheduling with pilotage and tugging considerations[J]. Transportation Research Part E. 2021, 148: 1366–5545. doi: 10.1016/j.tre.2021.102231

[8] Jia S, Li S Q, Lin X D, Chen X H. Scheduling tugboats in a seaport[J]. Transportation Science, 2022,55(6): 1370–1391. doi: 10.1287/trsc.2021.1079

[9] Liu Z. Hybrid evolutionary strategy optimization for port tugboat operation scheduling. 3rd International Symposium on Intelligent Information Technology Application, Nanchang, China, 21–22 December 2009. doi: 10.1109/IITA.2009.490

[10] Xu Q, Mao J, Jin Z H. Simulated Annealing-Based Ant Colony Algorithm for Tugboat Scheduling Optimization[J]. Mathematical Problems in Engineering, 2012: 246978. doi: 10.1155/2012/246978

[11] Kang L, Meng Q, Tan K C. Tugboat scheduling under ship arrival and tugging process time uncertainty[J]. Transportation Research Part E: Logistics and Transportation Review, 2020, 144: 102125. doi: 10.1016/j.tre.2020.102125

[12] Wang X, Liang Y J, Wei X Y, Chew E P. An adaptive large neighborhood search algorithm for the tugboat scheduling problem[J]. Computers & Industrial Engineering, 2023, 177: 109039. doi: 10.1016/j.cie.2023.109039

[13] Goli A, Ala A, Hajiaghaei-Keshteli M. Efficient multi-objective meta-heuristic algorithms for energy-aware non-permutation flow-shop scheduling problem[J]. Expert Systems with Applications,2023, 213: 119077. doi: 10.1016/j.eswa.2022.119077

[14] Tirkolaee E B, Goli A, Gutmen S, Weber G W, Szwedzka K. A novel model for sustainable waste collection arc routing problem: Pareto-based algorithms[J]. Annals of Operations Research, 2023, 324: 189–214. doi: 10.1007/s10479-021-04486-2 35068644 PMC8765820

[15] Tirkolaee E B, Goli A, Mardani A. A novel two-echelon hierarchical location-allocation-routing optimization for green energy-efficient logistics systems[J]. Annals of operations research, 2023, 324: 795–823. doi: 10.1007/s10479-021-04363-y

[16] Moharam R, Ali A F, Morsy E, Ahmed MA; Mostafa M-S M. A Discrete Chimp Optimization Algorithm for Minimizing Tardy/Lost Penalties on a Single Machine Scheduling Problem[J]. IEEE Access, 2022, 10: 52126–52138. doi: 10.1109/ACCESS.2022.3174484

[17] Zhong H, Zhang Y G, Gu Y M. A Bi-objective green tugboat scheduling problem with the tidal port time windows[J]. Transportation Research Part D-Transport and Environment, 2022, 110. doi: 10.1016/j.trd.2022.103409

[18] Mirjalili S, Mirjalili S M, Lewis A. Grey wolf optimizer[J]. Advances in Engineering Software, 2014,69: 46–61. doi: 10.1016/j.advengsoft.2013.12.007

[19] Komaki G M, Kayvanfar V. Grey Wolf Optimizer algorithm for the two-stage assembly flow shop scheduling problem with release time[J]. Journal of computational science, 2015, 8:109–120. doi: 10.1016/j.jocs.2015.03.011

[20] Fountas N A, Papantoniou I, Kechagias J D, Manolakos D E, Vaxevanidis N M. Modeling and optimization of flexural properties of FDM-processed PET-G specimens using RSM and GWO algorithm[J]. Engineering failure analysis, 2022, 138. doi: 10.1016/j.engfailanal.2022.106340

[21] Ambika Virupakshappa, Lim S J. Hybrid image embedding technique using Steganographic Signcryption and IWT-GWO methods[J]. Microprocessors and Microsystems, 2022(95): 104688. doi: 10.1016/j.micpro.2022.104688

[22] Ghalambaz M, Yengejeh R J, Davami A H. Building energy optimization using Grey Wolf Optimizer (GWO)[J]. Case Studies in Thermal Engineering, 2021, 27(4): 101250. doi: 10.1016/j.csite.2021.101250

[23] Jordehi A R. Optimal scheduling of home appliances in home energy management systems using grey wolf optimisation (GWO) algorithm[C]// 2019 IEEE Milan PowerTech,2019. doi: 10.1109/PTC.2019.8810406

[24] Liang X, Wang D, Huang M. Improved Grey Wolf Optimizer and Their Applications[C]//2019 IEEE 7th International Conference on Computer Science and Network Technology (ICCSNT), 2019:107–110. doi: 10.1109/iccsnt47585.2019.8962504

[25] Zheng Q, Veeravalli B, Tham C K. On the design of fault-tolerant scheduling strategies using primary-backup approach for computational grids with low replication costs[J]. IEEE Transactions on Computers, 2009, 58(3): 380–393. doi: 10.1109/TC.2008.172

[26] Zuo W L, Gao Y L. Differential Evolution algorithm based on random neighborhood variation and optimal reverse learning [J]. Application Research of Computers,2023,40(7): 1–12. doi: 10.19734/j.issn.1001-3695.2022.11.0785

[27] Yu H F. Evaluation of cloud computing resource scheduling based on improved optimization algorithm[J]. Complex & Intelligent Systems,2021,7(4): 1817–1822. doi: 10.1007/s40747-020-00163-2

[28] Hou Y X, Gao H B, Wang Z J, Du C S. Improved Grey Wolf Optimization Algorithm and Application[J]. Sensors,2022,22(10): 3810. doi: 10.3390/s22103810 35632219 PMC9147573

[29] Meng X J, Ma X L, Guan Z F. Optimization of SVM transformer fault diagnosis by multi-strategy improved Grey Wolf optimization algorithm[C]// 9th International Forum on Electrical Engineering and Automation (IFEEA),2022,1163–1169. doi: 10.1109/IFEEA57288.2022.10038252

[30] Tizhoosh H R. Opposition-based learning: a new scheme for machine intelligence[C]// International Conference on Computational Intelligence for Modelling, Control and Automation/International Conference on Intelligent Agents Web Technologies and International Commerce, 2005: 695–701. doi: 10.1109/cimca.2005.1631345

[31] Li L G, Huang X W, Qian S Q, Li Z F, Li S J, Mansour R F. Fuzzy Hybrid Coyote Optimization Algorithm for Image Thresholding[J]. CMC-Computers Materials & Continua, 2022,72(2): 3073–3090. doi: 10.32604/cmc.2022.026625

[32] Ministry of Transportation and Communications. Circular of the National Development and Reform Commission on the Revision and Issuance of the Measures for the Billing of Port Charges. 2019 Mar13 [cited 20 Aug 2023] [Internet] China. Available from: https://xxgk.mot.gov.cn/2020/xzgfxwj/202303/t20230317_3776495.html.

