# Supplementary material for: An improved gray wolf optimization to solve the multi-objective tugboat scheduling problem
Source: PLoS One. 2024 Feb 26;19(2):e0296966. doi: 10.1371/journal.pone.0296966 (PMC10896540; doi:10.1371/journal.pone.0296966)
Supplement: S1 Appendix — The S1 Appendix describes the rules and regulations related to port billing in China. (DOCX) [file pone.0296966.s001.docx]

Port Charges Billing Methodology

**Chapter I. General provisions**

Article 1 In accordance with the overall deployment of the Third Plenary Session of the 18th CPC Central Committee on comprehensively deepening the reform, in order to regulate the behavior of port operation and service charges, improve the port price formation mechanism, safeguard the legitimate rights and interests of all parties involved in the operation, use and management of the port, and to promote the sustained and healthy development of the port business, in accordance with the Port Law of the People's Republic of China, the Price Law of the People's Republic of China, the Central Pricing Catalogue, and the Central Enterprise-Related Operation and Service Charges.

Article 2 The People's Republic of China coastal, the Yangtze River mainline major ports and all other ports open to the outside world, to provide ships in and out, berthing, berthing, passengers on and off, cargo loading and unloading, barging, storage and port security and other services, by the port operator and pilotage agencies, such as units of the port to the ship side, the cargo side, or their agents, such as port business service fees, shall be applicable to the present measures.

Port charges for transportation between various ports and Hong Kong, Macao and Taiwan shall be implemented in accordance with the relevant provisions of these Measures concerning ships sailing international routes and foreign trade import and export goods and containers.

The fee billing methods of other ports, which are formulated according to the pricing authority and specific scope of application stipulated in the local pricing catalog, may be implemented with reference to the relevant provisions of these Measures.

The fee billing method for pilotage (berthing) of vessels on the mainline of the Yangtze River is separately stipulated.

Article 3 The port charges include the operation service charges with government-priced, government-guided and market-adjusted prices, among which the port charges with government-priced prices include the cargo port charges and port facility security charges; the port charges with government-guided prices include the pilotage (berthing) charges, tugboat charges, berthing charges and oil boom usage charges; the port charges with market-adjusted prices include the port operation lump sum charges and storage yard usage charges, The port charges subject to market-adjusted prices include port operation lump sum fee, storage yard use fee, ship supply service fee, ship pollutant reception and treatment service fee, and cargo handling service fee.

The above charges shall be collected separately, and port operators and pilotage agencies shall not set up other port charges beyond the above scope.

Port operators and pilotage agencies to establish a fee catalog list system, take the public notice board, public notice board, price list (book) or electronic display screen, electronic touch screen and other ways to take the initiative to publicize the charges, the corresponding service content and fee standards, and accept social supervision. Charge notice board (including notice board, electronic display screen, electronic touch screen, etc.) should be long-term fixed in the charge place as well as the port area where it is convenient to read, as far as possible, independent placement, location is obvious, the font is correct and standardized.

Article 4 The implementation of government pricing of port charges must be charged in accordance with the provisions of these measures; the implementation of government-guided price of port charges should be prescribed by these measures for the upper limit of the charges, port operators and pilotage agencies can independently formulate specific charges within the scope of not exceeding the upper limit of the charges; market-adjusted price of port charges by the port operator according to the market supply, demand and competition, production and operation costs and service content of the independent development of charges. Port operators shall set their own charges according to the supply and demand in the market and the competitive situation, production and operation costs and service contents.

Port charges for which government pricing is implemented shall be determined in accordance with the rates stipulated in these Measures; port charges for which government-guided pricing is implemented shall be determined in accordance with the total of the base rate, additional charges and preferential charges stipulated in these Measures.

Pilotage (berthing) fees for specific charges shall be copied by the port administration department where the port is located to the provincial transportation and price authorities, by the pilotage agency announced to the public for implementation.

Article 5 The shipping party, the cargo party or its agent shall submit written information concerning the payer to the port operator, administrator or pilotage agency no later than the day the ship arrives at the port. If the import and export manifests and the relevant information provided by the ship party or its agent are incorrect or need to be changed, the ship party or its agent shall notify the port operator, administrator or pilotage agency in writing before discharging or loading the ship.

Article 6 The port charges billing unit and into the whole method shall comply with the following provisions:

(a) Fees are calculated in Renminbi Yuan. The final number of each item of each bill of lading or loading order shall be rounded up to the nearest whole number, and the minimum charge for each billing order shall be RMB 1 yuan.

(b) Ships are billed in terms of billable tons, calculated on the basis of net tons, with 1 net ton being 1 billable ton, gross tons if there are no net tons, deadweight tons if there are neither net nor gross tons, and displacement if there are neither net nor gross and deadweight tons, and all are billed at the rate of the billable ton. Tugs are calculated on the basis of horsepower, with 1 horsepower being 1 billable ton. Wooden and bamboo rafts, floating objects, etc. are calculated by volume, and 1 cubic meter is 1 billable ton. If the volume is less than 1 billable ton, it will be charged at the rate of 1 billable ton.

(c) Time is billed in days or hours. If the billing unit is a day, it shall be counted as a calendar day, and if it is less than one day, it shall be counted as one day; if the billing unit is an hour, it shall be counted as one hour if it is less than one hour, and if it exceeds the end of one hour, it shall be counted as 0.5 hour if it is less than half an hour, and if it exceeds half an hour, it shall be counted as one hour. Except as otherwise provided.

(d) Distances are measured in nautical miles or kilometers, and if less than 1 nautical mile or 1 kilometer, 1 nautical mile or 1 kilometer.

(e) The area is billed in square meters, and if it is less than 1 square meter, it is billed as 1 square meter.

(f) Cargo is billed in weight tons or volume tons, and if there are both weight tons and volume tons, the greater is billed. Weight tons for the gross weight of the goods to 1000 kg for 1 weight tons; volume tons for the goods "full foot measurement" of the volume to 1 cubic meter for 1 volume tons. The weight of special cargo is converted according to Table 1 (special cargo weight conversion table), and when the actual weight is heavier than the converted weight, it is calculated according to the converted weight.

(vii) The weight or volume of each item of cargo on each bill of lading or loading order shall be calculated as a minimum of 1 weight ton or 1 volume ton; if it exceeds 1 weight ton or 1 volume ton, the balance shall be rounded up to the nearest 0.01. Goods of the same class in each bill of lading shall be rounded up.

(viii) Containers are billed in boxes (20 feet or 40 feet). Empty collapsible containers, 4 boxes and 4 boxes or less stacked together, according to 1 box of the corresponding standard heavy box billing. Except as otherwise provided.

Article 7 The weight or volume of the goods shall be as stated in the bill of lading, loading note or contract for the operation of port cargo. The port operator or manager may verify the weight or volume of the goods, and if the weight or volume listed on the bill of lading, loading note or contract for the operation of port cargoes does not conform to the verification, the actual verification results shall be used as the basis for billing.

Article 8 In addition to cargo harbor fees and port facility security fees, pilotage (berthing) fees, tugboat fees, berthing fees and oil boom usage fees shall be charged at the upper limit of the fees stipulated in these measures.

**Chapter II. Cargo port charges**

Article 9 The goods and containers through the port throughput, by specifically responsible for the maintenance and management of breakwaters, fairways, anchorages and other port infrastructure units to collect cargo port charges to the cargo side or its agent.

Article X. The collection of port charges for foreign trade goods shall be in accordance with the following provisions:

(i) Port charges for foreign trade cargo are charged at the rates specified in Table 2 (Rate Table for Port Charges for Foreign Trade Cargo) for inbound and outbound cargo respectively.

(ii) The following goods and containers are exempted from port charges for foreign trade cargo:

1. Baggage checked in on the basis of a passenger ticket;

2. Fuel materials for the ship's own use;

3. Ship's loading pad bondage material;

4. Packing spares accompanying the packaged goods;

5. Ice and salt for preservation that comes with the fish;

6. Necessary feed to accompany live animals and birds;

7. Embassy items, United Nations items, complimentary gifts, exhibits, samples;

8. International transit goods;

9. Empty containers (except commodity containers).

Article 11 The collection of port charges for domestic trade goods shall be in accordance with the following provisions:

(a) Domestic cargo port charges are collected at the rates specified in Table 3 (Domestic Cargo Port Charges Rate Table) for inbound and outbound cargo respectively.

(ii) The following goods and containers are exempted from port charges for domestic trade goods:

1. Baggage checked in on the basis of a passenger ticket;

2. Fuel materials for the ship's own use;

3. Ship's loading pad bondage material;

4. Packing spares accompanying the packaged goods;

5. Fresh fish caught by fishing boats as well as ice and salt used for preservation by their counterparts;

6. Necessary feed to accompany live animals and birds;

7. Embassy goods, United Nations goods, military goods;

8. Goods temporarily unloaded in the port due to accidents and still need to be transported to the original port of arrival;

9. Goods for the construction of Hong Kong;

10. Ships purchased or sold;

11. Empty containers (except commodity containers).

**Chapter III. Port Facility Security Fees**

Article 12 For foreign trade import and export goods and containers through the port, the port operator who has obtained the Certificate of Conformity of Port Facility Security shall charge the cargo party or its agent the inbound and outbound port facility security charges according to the rates stipulated in Table 4 (Port Facility Security Charge Rate Table).

Article XIII of foreign trade into and out of the internal feeder transportation containers, by undertaking international transportation section of the ship or its agent to the port of call port operator to pay port facilities security fees.

Article XIV foreign trade imported goods and containers for any reason to stay in the port of transit and no longer through the water transport to the port of arrival or other ports, port facilities security fees charged by the port of transit; for any reason to stay in the port of transit did not handle customs clearance procedures and continue to travel by water to the original port of arrival or other ports, port facilities security fees charged by the port of arrival.

Article 15 The following goods and containers are exempted from port facility security charges:

1. Baggage checked in on the basis of a passenger ticket;

2. Fuel materials for the ship's own use;

3. Ship's loading pad bondage material;

4. Packing spares accompanying the packaged goods;

5. Ice and salt for preservation that comes with the fish;

6. Necessary feed to accompany live animals and birds;

7. Embassy items, United Nations items, complimentary gifts, exhibits, samples;

8. Import of fertilizers, international transit and international transit goods and containers;

9. Empty containers (including commodity containers).

**Chapter IV. Pilotage (Mooring) Fees**

Article 16 The collection of pilotage fee from the vessel or its agent for leading a ship sailing an international route into or out of port shall be in accordance with the following provisions:

(a) Pilotage fees for pilotage distance of 10 nautical miles or less and for leading a ship of 120,000 net tons or less shall be charged at the rate specified in Table 5 (Port Charges for Vessels Engaged in International Routes) No. 1 (A). Pilotage fee is charged at 49,000 Yuan for the pilotage distance of 10 nautical miles or less and the pilotage fee for the vessel exceeding 120,000 net tons.

(ii) Pilotage charges for pilotage distances in excess of 10 nautical miles shall be charged at the rates specified in Table 5, No. 1 (B) for the excess distance.

(c) For pilotage charges beyond the pilotage distance of each port, the pilotage charges for the excess portion shall be charged at 30% of the rate specified in Table 5, No. 1 (A).

(D) Dalian, Yingkou, Qinhuangdao, Tianjin, Yantai, Qingdao, Rizhao, Lianyungang, Shanghai, Ningbo, Xiamen, Shantou, Shenzhen, Guangzhou, Zhanjiang, Fangchenggang, Haikou, Yangpu, Basho, Sanya ports other than ports (port areas), the pilotage fee plus a pilotage surcharge, the maximum maximum of not more than 0.27 yuan per billable ton.

(e) To lead the navigation of international route ships through the gate, the pilotage fee is added to the gate leading fee, and the gate leading fee is charged according to the rate stipulated in Table 5, No. 1 (C).

Article 17 The pilotage fee charged to the ship or its agent for leading a ship on a domestic voyage into or out of port shall be in accordance with the following provisions:

(i) Pilotage charges for pilotage distances of 10 nautical miles or less shall be charged at the rates specified in Table 6 (Base Rate Table for Port Charges for Vessels Engaged on Domestic Routes) No. 1 (A).

(ii) Pilotage charges for pilotage distances in excess of 10 nautical miles shall be charged at the rates specified in Table 6, No. 1 (B) for the excess distance.

(iii) For pilotage charges beyond the pilotage distance of each port, the pilotage charges for the excess portion shall be charged at 30% of the rate specified in Table 6, No. 1 (A).

Article 18 The pilotage distance of the port by the port administration where the port to determine and publicize, while copying the provincial transport authorities.

Article 19 The pilotage agency shall collect berthing fee from the vessel or its agent for leading international and domestic ships to move within the harbor. If a ship leading an international voyage moves within the harbor, the berthing fee shall be charged according to the rate stipulated in No. 1 (D) of Table 5 (Base Rate Table of Port Charges for Ships Engaged in International Voyages). If the vessel is guided to move within the harbor for domestic voyages, the berthing fee shall be charged according to the rate specified in Table 6 (Port Charges for Vessels Engaged in Domestic Vessels), No. 1 (C).

Article 20 For ships navigating domestic routes through the Heilongjiang water system, the pilotage fee shall be charged according to the rates specified in Table 7 (Benchmark Rate Table for Pilotage Fee for Ships Navigating Domestic Routes through the Heilongjiang Water System), and the pilotage fee for ships of more than 20,000 dues-paying tons and ships of more than 4,000 dues-paying tons towed by tugboats towing barges, wooden and bamboo rafts, and floating objects in water shall be determined by the negotiation between the pilotage authority and the ship or its agent; and the fee for moving berths within the harbor shall be charged according to the rates specified in Table 6 (Benchmark Rate Table for Port Charges for Ships Navigating Domestic Routes) No. 1 (D). The port shifting fee is charged according to the rate specified in Table 6 (Table of Port Charges for Vessels Sailing Domestic Routes) No.1 (D).

Article 21 Pilotage (berthing) operations on holidays or night shifts of ships navigating international routes shall be subject to additional surcharges on pilotage (berthing) fees in accordance with the actual operating conditions. If the operation time of pilotage (berthing) on holidays or night shift accounts for half or more of the total operation time, or if the operation time of holidays or night shift is more than or equal to half an hour, the surcharge for pilotage (berthing) fee for holidays or night shift shall be increased by 45% of the rate specified in Table 5 (Port Charges for Vessels Sailing International Routes Benchmark Rate Table) No.1, and the surcharge for the pilotage (berthing) fee for both holidays and night shift shall be increased by 90% of the rate specified in Table 5 (Port Charges for Vessels Sailing International Routes Benchmark Rate Table) No. 1. The surcharge for pilotage (berthing) fee for both holidays and night shifts shall be added at 90% of the rate specified in Table 5, No. 1.

Article 22 The minimum billable tons of port pilotage (berthing) for ships navigating international routes is 2,000 billable tons; the minimum billable tons of port pilotage (berthing) for ships navigating domestic routes is 300 billable tons for ships navigating the Heilongjiang water system, and the minimum billable tons of port pilotage (berthing) for ships navigating domestic routes is 500 billable tons for other ships navigating domestic routes.

Article 23 Pilotage fees are charged once for the first entry and once for the last exit.

Article 24 by the tugboat towing ships, barges, wood and bamboo rafts or floating objects on the water, the pilotage (berthing) fee according to the tugboat's power (horsepower) and the towed ships, barges, wood and bamboo rafts or floating objects on the water of the total billing tons charged.

**chapter v. tugboat charges**

Article 25 If a ship uses tugboats to berth or leave a ship and uses tugboats to pilot or move a ship, the unit providing tugboat service shall charge the ship or its agent for the tugboat fee. The rate per tugboat trip for ships navigating international and domestic routes shall be charged in accordance with the provisions of Table 8 (Tugboat Fee Benchmark Rate Table for Ships Navigating International Routes), Table 9 (Tugboat Fee Benchmark Rate Table for Ships Navigating Domestic Routes for Coastal Harbors) and Table 10 (Tugboat Fee Benchmark Rate Table for Ships Navigating Domestic Routes for Inland Waterway Harbors), respectively.

The standards for the number of tugboats to be used for berthing, de-berthing and piloting or shifting of ships in coastal ports are proposed by the local port administrative departments together with the maritime administration agencies, and the competent provincial transportation departments will review and approve the compliance and reasonableness of the standards and make them public. The standard for the number of tugboats on the mainline of the Yangtze River shall be formulated by the Yangtze River Shipping Administration of the Ministry of Transportation and Communications in conjunction with the relevant provincial transportation authorities along the river, and shall be announced to the public.

Article 26 If the distance between the berth from which the towed ship is pulling out and the nearest tug base is more than 30 nautical miles but less than or equal to 50 nautical miles, its towage fee may be charged at 110% of the base rate; if the distance is more than 50 nautical miles, it may be charged at 120%.

Article 27 The tugboat fee and fuel prices are linked, and when fuel prices rise or fall significantly affecting the operating costs of tugboats, the base rate of the tugboat fee will be adjusted appropriately. The specific linkage mechanism and methods are separately stipulated.

**Chapter VI. Parking fees**

Article 28 For ships moored at port terminals and floats, the port operator providing mooring services shall collect mooring fees from the ships or their agents. The collection of berthing fees shall be in accordance with the following provisions:

(a) For ships sailing international and domestic routes, berthing fees are charged at the rates specified in Table 5 (Base Rate Table for Port Charges for Ships Sailing International Routes) No. 2(A) and Table 6 (Base Rate Table for Port Charges for Ships Sailing Domestic Routes) No. 2(A) respectively.

(ii) The following ships sailing international and domestic routes shall be charged berthing fees at the rates specified in Table 5, No. 2 (B) and Table 6, No. 2 (B) respectively:

1. Vessels that remain at anchor for reasons attributable to the vessel four hours after the loading and unloading of cargo and containers or the embarkation and disembarkation of passengers has been completed;

2. Vessels undergoing repair or overhaul for reasons other than port reasons (except for repair or overhaul in the course of loading, unloading, loading and unloading of cargoes and containers);

3. Vessels remaining at anchor after refueling and watering;

4. Vessels not loaded or unloaded by port workers;

5. International passenger and tourist ships.

Article 29 For ships sailing international routes berthed in port anchorage, the unit responsible for maintaining the port anchorage shall collect berthing fees from the ship party or its agent according to the rate stipulated in No. 2 (C) of Table 5 (Base Rate Table of Port Charges for Ships Sailing International Routes).

Article 30 The berthing of a ship at a pier, float or anchorage in a harbor shall be 24 hours for one day, and if it is less than 24 hours, it shall be counted as one day. If a ship crosses the dock, float or anchorage in the harbor every 24 hours, the mooring fee shall be charged according to the rate stipulated in Table 5 No. 2 (A).

Article 31 A ship berthed to a ship moored to a pier or float in a harbor shall be charged mooring fees as if it were a ship moored to a pier or float.

Article 32 The berthing fee shall be waived for ships staying in port due to port reasons or special meteorological reasons, as well as for ships engaged in port construction works, military ships and official ships carrying out official duties.

**Chapter VII. Boom user fees**

Article 33 Ships shall use oil booms in accordance with regulations, and the unit providing oil boom services shall collect oil boom usage fees from the obligor of laying oil booms specified in the relevant regulations.

Article 34 The fuel oil containment fee for ships navigating international routes shall be charged at the rate specified in Table 5 (Base Rate Table of Port Charges for Ships Navigating International Routes) No. 3. Bunker usage charges for ships sailing on domestic routes shall be charged at the rates specified in Table 6 (Base Rate Table for Port Charges for Ships Sailing on Domestic Routes) No. 3.

**chapter viii. port operation lump sums**

Article 35 The port operator shall provide port loading and unloading and other labor operations for goods and containers transported by ships, and collect port operation lump-sum fees from ships, cargo parties or their agents, etc.; the port operator shall provide services such as the use of port and station for passenger and tourist ships, and collect port operation lump-sum fees from passenger and tourist ship operating enterprises or their agents.

Article 36 The scope of the port operation lump sum fee includes the whole process of port operation, and the port operator shall include the following cargo and container port operation and passenger port service in the port operation lump sum fee respectively, and shall not set up separate charges for separate items:

(i) Cargo and container port operations: breakbulk cargo handling (including leveling for the addition of cargo to the bulk of the ship's hold, as well as other special leveling as required by the ship or its agent), container loading and unloading, the use of railroad lines, railroad wagons to pick up and deliver, automobile loading and unloading, removal, reloading, container trains, barge loading and unloading (including the use of tugboats in the Yangtze River trunk line and the Heilongjiang River system ports to pick up and deliver the barge to the terminal for loading and unloading of cargoes) Container unloading, loading, lifting ships, cranes, crane use, lifting machine labor, unpacking and dumping packages, filling and sewing packages, split tickets, picking samples, general sweeping and removal of bulkheads, loading and unloading of rain equipment, the use of rain cover, loading and unloading and other operational hours, the use of shore machine, as well as difficult work, miscellaneous operations, load shedding, pounding loads, transferring the stacks, the operation of extra-long (bulky, hazardous, refrigerated, sporadic) cargoes, the weighing scale Use, rail weighing, measuring, use of elevators or other machinery in the warehouse, dust removal, container cleaning, use of tools in groups.

(ii) Passenger port services: passenger and tourist terminal services, port station use services, baggage agents, baggage loading and unloading, and welcoming passengers to and from terminals.

Article 37 The port operator may increase or reduce the operation contents stipulated in Article 36 according to the port operation situation, but they shall be included in the unified charging of the port operation lump sum fee, and the charging standard shall be independently formulated by the port operator.

Article 38 The port operation package fee shall not include the implementation of government pricing, government-guided price charges and other market-adjusted price charges.

**Chapter IX. Charges for the use of depots**

Article 39 The goods and containers are stored in port warehouses and yards, or, with the consent of the port operator, are processed and sorted, sampled, etc., in the port depot, and the port operator collects depot usage fees from the cargo party or its agent.

Article 40 The charges for the use of the depot shall be set by the port operator on its own.

**Chapter X. Fees for ship supply services and for services for the reception and treatment of pollutants from ships**

Article 41 The provision of water (materials), oil (gas), shore power and other supply services for ships, the unit providing the services to the ship or its agent to collect ship supply service fees.

Article 42 Provision of ship pollutant reception and treatment services such as garbage reception and treatment, oil and grease water reception and treatment, etc., for ships shall be subject to the collection of ship pollutant reception and treatment service fees by the unit providing the services from the ship or its agent.

Article 43 Charges for ship supply services and ship pollutant reception and treatment services shall be set independently by the units providing the services. The prices of water, oil, gas and electricity shall be implemented in accordance with the price policies stipulated by the State.

**Chapter XI Bylaws**

Article 44 The measures referred to in the "full-foot measurement" refers to "import and export commodities load measurement test procedures" (SN/T 0892) for measurement.

Article 45 The "dangerous goods" referred to in these Measures shall be implemented in accordance with the Provisions on Safety Management of Dangerous Goods in Ports (Decree of the Ministry of Transportation and Communications No. 27 of 2017).

Article 46 The holidays referred to in these Measures refer to the legal holidays and vacation days of the People's Republic of China. Night shift operation time refers to the operation time of 8 consecutive hours from 21:00 to 08:00 of the next day, and the specific starting and ending points of the time shall be determined by the port administration department where the port is located and announced to the public.

Article 47 For coastal container ships on domestic feeder routes, port charges with government-guided pricing shall be charged at 50% of the rates stipulated in Table 5 (the table of benchmark rates for port charges for ships navigating international routes); for container ships on domestic feeder routes of the Yangtze River, port charges with government-guided pricing shall be charged at the rates stipulated in Table 6 (the table of benchmark rates for port charges for ships navigating domestic routes); and for ships that are carrying marine crude oil, liquefied petroleum gas (except for foreign trade), import and export crude oil and liquefied petroleum gas, port charges shall be charged at 50% of the rates stipulated in Table 5. For ships carrying marine crude oil and liquefied petroleum gas (except foreign trade crude oil and liquefied petroleum gas), the port charges under government-guided pricing shall be charged at 50% of the rates stipulated in Table 5.

Article 48 Port operation charges for the transportation of rescue and disaster relief materials shall be formulated by the Ministry of Transport in conjunction with the National Development and Reform Commission. Port operation charges for military transportation shall be formulated by the Ministry of Transport in conjunction with the administrative department responsible for military transportation and the National Development and Reform Commission.

Article 49 These measures shall be interpreted by the Ministry of Transport in conjunction with the National Development and Reform Commission.

Article 50 These Measures shall be implemented as of April 1, 2019, and shall be effective for a period of five years.On July 12, 2017, the Measures on Port Charge Billing issued by the Ministry of Transport and the National Development and Reform Commission ceased to be implemented. Where the relevant provisions previously issued are inconsistent with these Measures, these Measures shall prevail.

**Table 1 Conversion table for special cargo weights**

| **Name of goods** | **unit of measure** | **Conversion weight (kg)** |
| --- | --- | --- |
| Camels, oxen, horses, mules, donkeys | remnant | 1000 |
| Pigs, sheep, dogs, calves, foals, mules and donkeys | Head (article, only) | 200 |
| Bulk piglets, lambs | Heads (only) | 30 |
| Caged piglets, lambs, poultry, livestock, wild animals, snakes, eggs | cubic meter (unit of volume) | 500 |
| Rattan, bamboo chairs, stools, tables, bookshelves | classifier for individual things or people, general, catch-all classifier | 30 |
| Fish fry (seedlings, seeds) | cubic meter (unit of volume) | 800 |
| Other goods whose weight cannot be determined | cubic meter (unit of volume) | 1000 |
| Furniture (except folded) | Twice the dead weight. | |
| Empty containers of various materials (except folded and straw, cloth, paper, sacks, plastic bags) |  |  |

Note: Twice the deadweight is the gross weight of the goods themselves plus twice that amount.

**Table 2 Scale of port dues for foreign trade cargoes**

| **categorization** | **serial number** | **Cargo and container name** | **billing unit** | **Rate ($)** | |
| --- | --- | --- | --- | --- | --- |
|  |  |  |  | **inlet** | **exportation** |
| merchandise | 1 | Coal, ores, mineral sands, mineral powders, phosphate ash, cement, soda ash, grain, salt, sand, stone, bricks and tiles, pig iron, steel (excluding scrap), steel pipes, steel billets, steel ingots, non-ferrous metal lump ingots, coke, semi-coke, lump coal, fertilizers, and light blister goods | dead weight ton | 1.20 | 0.60 |
|  |  |  | kiloton (unit of volume) | 0.70 | 0.35 |
|  | 2 | Class I dangerous goods, refrigerated goods, ancient paintings, antiques, goldware, silverware, jewelry, jade, jadeite, coral, agate, crystal, diamonds, jade carvings, wood carvings, all kinds of sculpture products, shellfish carvings, lacquer ware, antique porcelain, cloisonné, carpets, tapestries, embroidery | dead weight ton | 5.60 | 2.80 |
|  |  |  | kiloton (unit of volume) | 3.70 | 1.85 |
|  | 3 | Other goods | dead weight ton | 2.80 | 1.40 |
|  |  |  | kiloton (unit of volume) | 1.80 | 0.90 |
| container (for shipping) | 4 | Containers, commodity containers for general cargo | Box (20 feet) | 34.00 | 17.00 |
|  |  |  | Box (40 feet) | 68.00 | 34.00 |
|  | 5 | Containers, reefer containers (heavy lift) containing dangerous goods of Class I | Box (20 feet) | 68.00 | 34.00 |
|  |  |  | Box (40 feet) | 136.00 | 68.00 |

Note: 1. "Light foam cargo" means cargo with a volume of 4 cubic meters or more per 1 heavy ton, but if the weight of each piece of cargo is 5 tons or more, it shall be charged according to the weight ton.

2. "Fertilizers" in No. 1 refers to fertilizers used for agricultural production, except those used as chemical raw materials.

3.编号2中的“一级危险货物”包括《危险货物品名表》（GB12268）和《国际海运危险货物规则》（IMDG Code）危险货物一览表中的第1类、第2类、第7类、第5.2项和第6.2项的危险货物以及第3类、第4类、第 8, Division 5.1 and Division 6.1, excluding fertilizers and pesticides for agricultural production.

4. Crude oil is billed as "other goods" under No. 3.

5. Other containers in accordance with its internal volume and the proportion of the content of the container volume of the similar box type listed in the table billing.

**Table 3 Scale of port dues for domestic trade cargoes**

| serial number | categorization | Scope of application | billing unit | Rate ($) |
| --- | --- | --- | --- | --- |
| 1 | merchandise | coastal port | dead weight ton | 0.425 |
|  |  | river port |  | 0.85 |
|  |  | coastal port | kiloton (unit of volume) | 0.21 |
|  |  | river port |  | 0.42 |
| 2 | Containers, commodity containers for general cargo | Coastal and river ports | Box (20 feet) | 7.00 |
|  |  |  | Box (40 feet) | 14.00 |
| 3 | Containers, reefer containers (heavy lift) containing dangerous goods of Class I | Coastal and river ports | Box (20 feet) | 14.00 |
|  |  |  | Box (40 feet) | 28.00 |

Note: 1. Other containers are billed according to the ratio of their inner volume to the inner volume of containers of similar box types listed in the table.

2. Fuzhou Port is billed according to the inland waterway port charges.

**Table 4 Scale of Port Facility Security Charges**

| serial number | categorization | billing unit | Rate ($) |
| --- | --- | --- | --- |
| 1 | Container Heavy Duty | Box (20 feet) | 8.00 |
|  |  | Box (40 feet) | 12.00 |
| 2 | merchandise | Weight tons or volume tons | 0.20 |

Note: 1. Non-standard containers other than 20-foot heavyweight containers and 40-foot heavyweight containers will be billed at the rate of similar container type.

2. Containerized LCL cargo is assessed port facility security charges based on the actual weight tons or volume tons of the cargo.

**Table 5 Benchmark rates for port charges for ships plying international routes**

| **serial number** | **sports event** | **billing unit** | **Rate ($)** | | **clarification** |
| --- | --- | --- | --- | --- | --- |
| 1 | Pilotage (mooring) charges | billable ton | A | 0.45 | 40,000 net tons and below portion |
|  |  |  |  | 0.40 | 40,001-80,000 net tons portion |
|  |  |  |  | 0.375 | 80,000-120,000 net tons portion |
|  |  | Billable tons-nautical miles | B | 0.004 | Over-range portion above 10 nautical miles |
|  |  | billable ton | C | 0.14 | Leading through the gate |
|  |  | billable ton | D | 0.20 | In-port movement |
| 2 | parking fees | Billable ton-day | A | 0.25 |  |
|  |  | Billable ton-hours | B | 0.15 |  |
|  |  | Billable ton-day | C | 0.05 | anchor |
| 3 | Boom usage fees | Ship - times | 3000.00 | | Ships under 1000 net tons |
|  |  |  | 3500.00 | | Ships of 1000-3000 net tons |
|  |  |  | 4000.00 | | Ships over 3,000 net tons |

**Table 6 Benchmark rates for port charges for ships plying domestic routes**

| **serial number** | **sports event** | **billing unit** | **Rate ($)** | | **clarification** |
| --- | --- | --- | --- | --- | --- |
| 1 | Pilotage (mooring) charges | billable ton | A | 0.18 |  |
|  |  | Billable tons-nautical miles | B | 0.0018 |  |
|  |  | billable ton | C | 0.135 | Leading domestic vessels to move berths in the harbor |
|  |  |  | D | 0.105 | Leading the domestic route ships navigating the Heilongjiang water system in the harbor to move the mooring |
| 2 | parking fees | Billable ton-day | A | 0.08 |  |
|  |  |  | B | 0.12 |  |
| 3 | Boom usage fees | Ship - times | 1000.00 | | Ships under 500 net tons |
|  |  |  | 1200.00 | | Ships of 500-1000 net tons |
|  |  |  | 1400.00 | | Ships over 1,000 net tons |

**Table 7 Benchmark rates for pilotage charges for vessels navigating domestic routes in the Heilongjiang water system**

| **Vessel type** | **Billing unit (billable tons)** | **Rate ($/km)** |
| --- | --- | --- |
| Passenger and cargo ships | Less than 300  300-500  500-1000  1000-2000  2000-3000  3000-5000  5000-7000  7000-10000  10,000-15,000  15000-20000 | 0.89  1.33  1.77  2.37  2.66  3.11  3.56  4.29  5.47  7.99 |
| Barges, wooden and bamboo rafts, floating objects on water | Less than 500 | 0.44 |
|  | 500-1000 | 0.59 |
|  | 1000-2000 | 0.67 |
|  | 2000-3000 | 0.74 |
|  | 3000-4000 | 0.89 |

Note: Pilotage miles are based on tariff miles.

**Table 8 Benchmark rate of tugboat charges for vessels traveling international routes Billing unit: RMB/tugboat** trip

| serial number | Length of ship (meters) | Vessel type | | |
| --- | --- | --- | --- | --- |
|  |  | Container ships,  Ro-Ro ships, passenger ships | Tankers, chemical tankers,  Liquefied gas carriers | Bulk carriers, general cargo ships and others |
| 1 | 80 and below | 6000 | 5700 | 5300 |
| 2 | 80-120 | 6500 | 7800 | 7400 |
| 3 | 120-150 | 7000 | 8500 | 8000 |
| 4 | 150-180 | 8000 | 10500 | 9000 |
| 5 | 180-220 | 8500 | 12000 | 11000 |
| 6 | 220-260 | 9000 | 14,000 | 13,000 |
| 7 | 260-275 | 9500 | 16000 | 14,000 |
| 8 | 275-300 | 10000 | 17000 | 15000 |
| 9 | 300-325 | 10500 | 18,000 | 16000 |
| 10 | 325-350 | 11000 | 18600 | 16500 |
| 11 | 350-390 | 11500 | 19600 | 17800 |
| 12 | 390- | 12000 | 20300 | 19600 |

**Table 9 Benchmark rate table for tugboat charges for domestic voyages (coastal ports) Billing unit: Yuan/tugboat ship**

| serial number | Length of ship (meters) | Vessel type | | |
| --- | --- | --- | --- | --- |
|  |  | Container ships,  Ro-Ro ships, passenger ships | Tankers, chemical tankers,  Liquefied gas carriers | Bulk carriers, general cargo ships and others |
| 1 | 80 and below | 3300 | 3300 | 3000 |
| 2 | 80-120 | 3700 | 4300 | 4100 |
| 3 | 120-150 | 4200 | 4900 | 4700 |
| 4 | 150-180 | 4500 | 6100 | 5100 |
| 5 | 180-220 | 4800 | 6900 | 6100 |
| 6 | 220-260 | 5200 | 8500 | 7500 |
| 7 | 260-275 | 5500 | 9500 | 8000 |
| 8 | 275-300 | 5700 | 10000 | 8500 |
| 9 | 300-325 | 6100 | 10500 | 9000 |
| 10 | 325-350 | 6400 | 10700 | 9400 |
| 11 | 350-390 | 6700 | 11300 | 9900 |
| 12 | 390- | 7100 | 11900 | 11300 |

**Table 10 Benchmark rate table for tugboat charges for domestic voyages (inland waterway ports) Billing unit: RMB/tugboat ship number**

| serial number | Length of ship (meters) | Vessel type | | |
| --- | --- | --- | --- | --- |
|  |  | Container ships,  Ro-Ro ships, passenger ships | Tankers, chemical tankers,  Liquefied gas carriers | Bulk carriers, general cargo ships and others |
| 1 | 80 and below | 4900 | 4700 | 4200 |
| 2 | 80-120 | 5400 | 6100 | 5800 |
| 3 | 120-150 | 5800 | 6900 | 6500 |
| 4 | 150-180 | 6500 | 8600 | 7300 |
| 5 | 180-220 | 6800 | 9900 | 9000 |
| 6 | 220-260 | 7300 | 11700 | 10600 |
| 7 | 260-275 | 7900 | 13300 | 11700 |
| 8 | 275-300 | 8200 | 14,000 | 12400 |
| 9 | 300-325 | 8700 | 14900 | 13100 |
| 10 | 325-350 | 9100 | 15400 | 13700 |
| 11 | 350-390 | 9600 | 16300 | 14700 |
| 12 | 390- | 10000 | 17100 | 16200 |

Note: Inland river ports in this table include Yangtze River mainline ports and other inland river ports open to the outside world.
